# baredSC: Bayesian approach to retrieve expression distribution of single-cell data

**DOI:** 10.1186/s12859-021-04507-8

**Published:** 2022-01-12

**Authors:** Lucille Lopez-Delisle, Jean-Baptiste Delisle

**Affiliations:** 1 EPFL SV ISREC UPDUB, 1015 Lausanne, Switzerland; 2 Département d’astronomie, Université de Genève, Chemin Pegasi 51, 1290 Versoix, Geneva, Switzerland

**Keywords:** scRNA-seq, Bayesian, MCMC

## Abstract

**Background:**

The number of studies using single-cell RNA sequencing (scRNA-seq) is constantly growing. This powerful technique provides a sampling of the whole transcriptome of a cell. However, sparsity of the data can be a major hurdle when studying the distribution of the expression of a specific gene or the correlation between the expressions of two genes.

**Results:**

We show that the main technical noise associated with these scRNA-seq experiments is due to the sampling, i.e., Poisson noise. We present a new tool named baredSC, for Bayesian Approach to Retrieve Expression Distribution of Single-Cell data, which infers the intrinsic expression distribution in scRNA-seq data using a Gaussian mixture model. baredSC can be used to obtain the distribution in one dimension for individual genes and in two dimensions for pairs of genes, in particular to estimate the correlation in the two genes’ expressions. We apply baredSC to simulated scRNA-seq data and show that the algorithm is able to uncover the expression distribution used to simulate the data, even in multi-modal cases with very sparse data. We also apply baredSC to two real biological data sets. First, we use it to measure the anti-correlation between *Hoxd13* and *Hoxa11*, two genes with known genetic interaction in embryonic limb. Then, we study the expression of *Pitx1* in embryonic hindlimb, for which a trimodal distribution has been identified through flow cytometry. While other methods to analyze scRNA-seq are too sensitive to sampling noise, baredSC reveals this trimodal distribution.

**Conclusion:**

baredSC is a powerful tool which aims at retrieving the expression distribution of few genes of interest from scRNA-seq data.

**Supplementary Information:**

The online version contains supplementary material available at 10.1186/s12859-021-04507-8.

## Background

The single-cell RNA sequencing (scRNA-seq) method allows to assess the transcriptome of individual cells [1]. Thanks to the decreasing cost and to the simplification of the protocols, this technique is more and more popular. One can distinguish two classes of methods: the plate-based methods and the droplet-based methods (also called microfluidic-based methods). The first ones often provide a better estimation of genes expression for individual cells, as the number of reads per cell is usually higher. However, the experimental procedure is more complicated and these methods typically generate data for hundreds of cells. The second ones are more widely used and provide usually sparser information (less reads per cell) but for a larger number of cells (typically 50 times more) [2]. As a consequence, droplet-based methods are often preferred when complex tissues are studied. Indeed, they allow to identify more precisely pools of cells with close cellular identity (clusters) [3]. Gene expression within clusters are then studied in more details which is not possible when the number of cells is too low. However, with droplet-based methods, the number of reads for a given cell is very low compared to the complexity of the transcriptome. Therefore, lowly expressed genes are not always detected, and if detected, their expression level is poorly constrained. The fact that a gene for a given cell is not detected while it is actually expressed is sometimes called ‘dropout’ [2, 4–7]. The origin and modeling of the ‘dropout’ phenomenon, and more generally of the observed variance in scRNA-seq is debated, as reflected by the number of different models used in tools dedicated to differential expression analysis [8]. Here, we show that Poisson noise due to sampling explains very well the observed variance (dropout included) in droplet-based scRNA-seq. We find no evidence of more complex noise sources, such as additional dropouts, negative binomial distribution, etc.

For most common applications of scRNA-seq (clustering and identification of gene markers, projection like tSNE or UMAP) the sparsity of the data does not have a strong impact [9]. However, the expression distribution of a given gene in a population of cells is difficult to estimate and its representation by a kernel density estimation (KDE) plot, like in the violin plot of Seurat [10] or Scanpy [11], can be misleading. Indeed, the sampling noise strongly spread the signal, and the cells with no count appear as an artificial homogeneous sub-population, mixing cells with low and no expression.

Gene correlation has been extensively studied in bulk RNA-seq in order to build regulatory networks [12]. A recent study using scRNA-seq discovered gene covariations involving microRNA [13]. miRNA are small RNAs which regulate gene expression by post-transcriptional processes [14]. Such results would be difficult to obtain with bulk RNA-seq, showing the power of this type of study in homogenous scRNA-seq. However, this study has been conducted with a plate-based method on a very homogeneous population (mouse embryonic stem cells). When using droplet-based data, the sparsity of the data is a major barrier to this type of analysis restricting it to genes with high expression.

Recently, [15] proposed a Bayesian normalization procedure called Sanity (SAmpling-Noise-corrected Inference of Transcription activitY). This method aims at correcting the counts of each cell from the sampling noise. The procedure is fast and can be applied before clustering and projection algorithms to improve their performance. It also prevents spurious correlation between pairs of genes. However, in order to efficiently perform these corrections, Sanity uses simplifying assumptions in the modeling of the genes’ expression distributions. Here, we also propose a Bayesian approach to disentangle the intrinsic variability in gene expressions from the sampling noise. However, instead of focusing on correcting the expression level of each cell, we aim at retrieving accurately the underlying expression distribution of the population of cells. Our tool, named baredSC (Bayesian Approach to Retrieve Expression Distribution of Single-Cell data), approximates the expression distribution of a gene by a Gaussian mixture model (GMM). This tool is dedicated to studies where the distribution of few genes needs to be estimated precisely. It can be used to retrieve the expression distribution of a single gene, but also to infer the joint distribution of two genes in order to study genetic interactions even when the gene expression level is low and thus the frequency of non-detection is high.

Using simulated data we show that it largely outperforms the classical density/violin representation and in most cases very accurately reproduces the original distribution, both in the one-dimensional and two-dimensional cases. We also show that when multi-modal distributions are simulated it gives more accurate results than Sanity. We also use real biological datasets to illustrate the power of baredSC to assess the correlation between genes or to reveal the multi-modality of a lowly expressed gene.

## Results

### Poisson distribution is a good approximation for droplet-based single-cell RNA-seq data

We focus our analysis on droplet-based scRNA-seq as this is where the sparsity of the data is a major issue. We first evaluate the variability coming from the technique itself, including all steps of the protocol from RNA to the gene count matrix. Indeed, the source of variability in droplet-based scRNA-seq is still debated. The most obvious source of noise comes from the sampling, i.e. the fact that only a subset of the whole transcriptome is sequenced. This sampling noise is especially strong for lowly expressed genes and when the total number of reads per cell is low. In addition to the sampling noise, some specific steps of the technique, like the capture of mRNA or the amplification steps, could possibly introduce variability and/or biases [16].

The most visible consequence of the noise is the so-called ‘dropout’ effect. [2] studied this phenomenon and concluded that the fraction of cells with no count was fully compatible with a noise following a negative binomial distribution. However, this analysis was mostly conducted ignoring the variability in the total number of counts per cell which has a non-negligible effect on the variability of the number of counts for a given gene. Nevertheless, in the Additional file 1: Fig. S1 of [2], the use of scaled data suggests that the simpler Poisson distribution could be sufficient to explain the number of dropouts.

Here, we reanalyze the same datasets as [2], to evaluate all the noise contributions (dropouts and more generally the variability), using scaled data. Following [2], we take advantage of published control datasets [17–20] as well as two real biological datasets provided by 10X genomics with cells from a mouse cell line (NIH3T3) and from a human one (HEK293T). In the control datasets, a homogeneous solution of RNA was used as input instead of a solution of single-cells. This allows to study the technical noise without any influence of the cell to cell variability. Indeed, in these datasets, each pseudo single-cell was a droplet of the same RNA solution. On the contrary, the 10X genomics cell lines are real biological datasets where we expect to find both technical variations and biological variations.

When studying gene expression, the quantity of interest is the number of transcripts coding for a given gene *g* in cell *i*. Unfortunately, this quantification is very difficult to obtain and one often focus instead on the proportion of transcripts coding of a given gene *g* out of all transcripts in the cell *i*. We denote by $\lambda _{i,g}$ this fraction. An obvious estimator of $\lambda _{i,g}$ is

1 $$\begin{aligned} X_{i,g} = \frac{k_{i,g}}{N_i}, \end{aligned}$$

where $k_{i,g}$ is the number of counts for the gene *g* in the cell *i* and $N_{i}$ is the total number of counts identified in the cell *i*. However, since $N_{i}$ is always much smaller than the total number of transcripts in the cell, $k_{i,g}$ is strongly affected by sampling noise. Rigorously speaking, in the absence of other sources of noise, $k_{i,g}$ follows a binomial distribution with parameters $N_i$ and $\lambda _{i,g}$. This distribution can actually be approximated by a Poisson distribution with parameter $N_i \lambda _{i,g}$ for sufficiently small $\lambda _{i,g}$ and large $N_i$. We thus have

2 $$\begin{aligned} {{\,\mathrm{{\mathbb {E}}}\,}}(X_{i,g}) = \frac{{{\,\mathrm{{\mathbb {E}}}\,}}(k_{i,g})}{N_{i}} = \lambda _{i,g}, \end{aligned}$$

where ${{\,\mathrm{{\mathbb {E}}}\,}}(X)$ is the expectation of *X*, and

3 $$\begin{aligned} {{\,\mathrm{var}\,}}(X_{i,g}) = \frac{{{\,\mathrm{var}\,}}(k_{i,g})}{N_{i}^2} = \frac{\lambda _{i,g}}{N_{i}} = \frac{{{\,\mathrm{{\mathbb {E}}}\,}}(X_{i,g})}{N_{i}}, \end{aligned}$$

where ${{\,\mathrm{var}\,}}(X)$ is the variance of *X*. If additional sources of noise affect the experiment, one would expect an increase in variance. For instance, [2] compared the Poisson distribution with the negative binomial distribution with the same expectation and a variance following

4 $$\begin{aligned} {{\,\mathrm{var}\,}}(k_{i,g}) = N_i \lambda _{i,g} + \Phi \left( N_i\lambda _{i,g}\right) ^2, \end{aligned}$$

where $\Phi$ is a new parameter which allows to account for an additional variance with respect to the Poisson distribution. In particular, the Poisson distribution is recovered for $\Phi =0$. In terms of $X_{i,g}$, the expectation still follows Eq. (2), and the variance is now written as

5 $$\begin{aligned} {{\,\mathrm{var}\,}}(X_{i,g}) = \frac{\lambda _{i,g}}{N_{i}} + \Phi \lambda ^2_{i,g}. \end{aligned}$$

For a given experiment, one only obtains a single realization of $X_{i,g}$, so the expectation and variance of $X_{i,g}$ cannot be measured in order to compare the Poisson and negative binomial distributions. However, in the case of control experiments, each pseudo single cell is sampled from the same RNA solution, so the fraction $\lambda _{i,g}$ is the same in each cell. We denote by $\lambda _g$ this common value. By combining the reads in all pseudo cells, one can determine a very precise estimate of $\lambda _g$

6 $$\begin{aligned} M_g = \frac{\sum _{i=1}^n k_{i,g}}{\sum _{i=1}^n N_i} = \frac{\sum _{i=1}^n N_i X_{i,g}}{\sum _{i=1}^n N_i}, \end{aligned}$$

which by construction verifies

7 $$\begin{aligned} {{\,\mathrm{{\mathbb {E}}}\,}}(M_g) = \lambda _g, \end{aligned}$$

for both the Poisson and negative binomial distributions. We additionally introduce the variance estimator

8 $$\begin{aligned} V_g = \frac{\sum _{i=1}^n N_i (X_{i,g} - M_g)^2}{\sum _{i=1}^n N_i}, \end{aligned}$$

which provides a measure of the spread (variance) of the $X_{i,g}$ values among the cells for a given gene *g*. The expected value of $V_g$ is

9 $$\begin{aligned} {{\,\mathrm{{\mathbb {E}}}\,}}(V_g) = \frac{n-1}{\sum _{i=1}^n N_i}\left( \lambda _g + \Phi \lambda _g^2 \frac{(\sum _{i=1}^n N_i)^2 - \sum _{i=1}^n N_i^2}{(n-1)\sum _{i=1}^n N_i}\right) . \end{aligned}$$

We define the normalized variance estimator

10 $$\begin{aligned} {\tilde{V}}_g = \frac{\sum _{i=1}^n N_i}{n-1} V_g \end{aligned}$$

such that

11 $$\begin{aligned} {{\,\mathrm{{\mathbb {E}}}\,}}({\tilde{V}}_g) = \lambda _g + \Phi \lambda _g^2 \frac{(\sum _{i=1}^n N_i)^2 - \sum _{i=1}^n N_i^2}{(n-1)\sum _{i=1}^n N_i}. \end{aligned}$$

In particular, in the case of the Poisson distribution ($\Phi =0$), we have ${{\,\mathrm{{\mathbb {E}}}\,}}({\tilde{V}}_g) = \lambda _g = {{\,\mathrm{{\mathbb {E}}}\,}}(M_g)$, while for $\Phi > 0$ (excess of variance) we obtain ${{\,\mathrm{{\mathbb {E}}}\,}}({\tilde{V}}_g) > {{\,\mathrm{{\mathbb {E}}}\,}}(M_g)$.

**Fig. 1:**
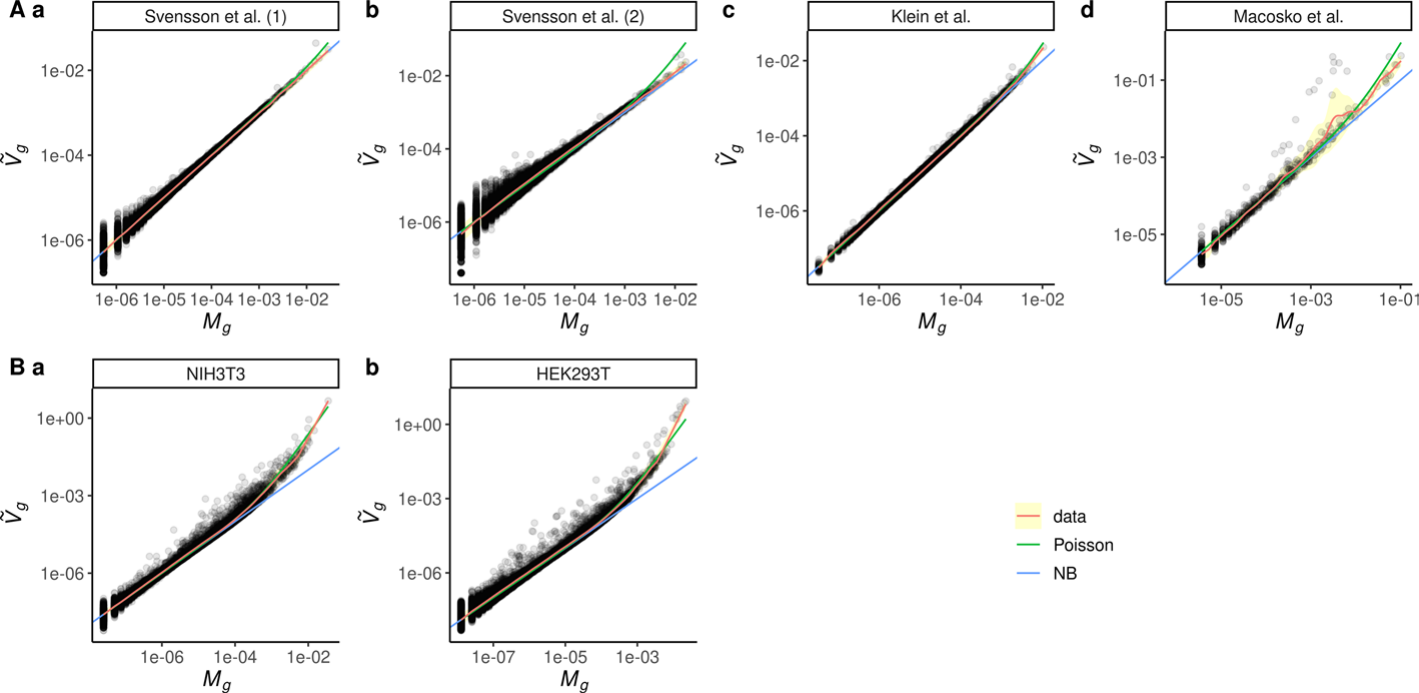
Poisson is a good approximation to explain variance in droplet-based single-cell RNA-seq. In each dataset, control dataset (**A**) or real single-cell experiment (**B**), the normalized variance estimator is plotted in function of the estimated mean expression. Each dot is a gene. A Gaussian kernel smoother was applied to evaluate the tendency of the data (red line) and the error around the Gaussian kernel smoothing was estimated (yellow area). The expected variance in the Poisson approximation is a straight line (blue) whereas the expected variance in the negative binomial approximation is a quadratic curve (green). Both axes are in log scale

To estimate which law is more adequate to explain the data we plot ${\tilde{V}}_g$ against $M_g$ for each gene *g* (Additional file 1: Fig. S1). If the data follow the Poisson distribution, we expect the points to align on the $y=x$ line, while if the data present an additional noise ($\Phi >0$), the points should end-up above this line. We noticed that the genes corresponding to ERCC (External RNA Control Consortium) spike-in had a behaviour different from what was observed for real genes, especially in the dataset from [18] where the variance is highly increased (Additional file 1: Fig. S1Ab). Thus, we decided to exclude these genes from the analysis. The results with ERCC spike-in information excluded are presented in Fig. 1. Overall, we find a very good agreement of the data with the Poisson distribution in the control datasets (Fig. 1A). In datasets from [20] (1) and (2) (Fig. 1Aa and b), the data perfectly follow the Poisson law for all expression levels. In the two other control datasets (from [17] and [18] Fig. 1Ac and d), both models perform equally well until a relatively high level of expression (about 1‰). Above 1‰, the variance slightly deviates from the Poisson prediction. However, in both cases, the deviation is very small and the results at high expression levels suffer from low number statistics. In addition, in the control experiment from [17], such expression corresponds to the top 0.5% highly expressed genes like ribosomal protein or cytoskeleton proteins. Therefore, we conclude that the Poisson law very well explains the variance observed in the control datasets.

On the contrary, when considering real experiments, even with a homogeneous single-cell population (Fig. 1B), we find that the variance strongly deviates from the Poisson prediction. Moreover, the deviation increases with the expression level and the negative binomial law captures this behaviour very well. This result clearly demonstrates the inter cellular heterogeneity in so-called homogeneous cell populations.

In [2], the author finds that the negative binomial distribution better matches the data than the Poisson law, even for control datasets. This seems to contradict our results. However, the analysis of [2] was performed on the raw number of counts $k_{i,g}$, without scaling it by the total number of counts $N_i$. The author uses the following estimators for the mean and variance of $k_{i,g}$

12 $$\begin{aligned} m_g&= \frac{1}{n} \sum _{i=1}^n k_{i,g},\nonumber \\ v_g&= \frac{1}{n-1} \sum _{i=1}^n (k_{i,g}-m_g)^2. \end{aligned}$$

If we assume $k_{i,g}$ to follow a Poisson distribution with parameter $N_i \lambda _g$, the expectation of $m_g$ and $v_g$ are written as

13 $$\begin{aligned} {{\,\mathrm{{\mathbb {E}}}\,}}(m_g)&= {\bar{N}} \lambda _g ,\nonumber \\ {{\,\mathrm{{\mathbb {E}}}\,}}(v_g)&= {\bar{N}} \lambda _g + {{\,\mathrm{var}\,}}(N)\lambda _g^2, \end{aligned}$$

where ${\bar{N}}$ is the average of the $N_i$ values, and ${{\,\mathrm{var}\,}}(N)$ their variance

14 $$\begin{aligned} {\bar{N}}&= \frac{1}{n} \sum _{i=1}^n N_i,\nonumber \\ {{\,\mathrm{var}\,}}(N)&= \frac{1}{n-1} \sum _{i=1}^n \left( N_i-{\bar{N}}\right) ^2. \end{aligned}$$

The variations of $N_i$ were neglected by [2], which would correspond to assuming $N_i = {\bar{N}}$ and ${{\,\mathrm{var}\,}}(N) = 0$ in Eq. (13). In this case, one would thus expect to find ${{\,\mathrm{{\mathbb {E}}}\,}}(m_g) = {{\,\mathrm{{\mathbb {E}}}\,}}(v_g) = {\bar{N}}\lambda _g$, if $k_{i,g}$ follows a Poisson distribution. Svensson [2] found the data to be better described by a negative binomial distribution for $k_{i,g}$ with a free parameters $\phi$ such that

15 $$\begin{aligned} {{\,\mathrm{{\mathbb {E}}}\,}}(m_g)&= {\bar{N}} \lambda _g ,\nonumber \\ {{\,\mathrm{{\mathbb {E}}}\,}}(v_g)&= {\bar{N}} \lambda _g + \phi \left( {\bar{N}}\lambda _g\right) ^2. \end{aligned}$$

This expression can actually match the formula we obtain assuming a Poisson distribution but accounting for the variability of $N_i$ (see Eq. (13)), if we take

16 $$\begin{aligned} \phi = \phi _\mathrm {Poisson} = \frac{{{\,\mathrm{var}\,}}(N)}{{\bar{N}}^2}. \end{aligned}$$

In Table 1, we compare the values of the parameter $\phi$ fitted by [2] with the values $\phi _\mathrm {Poisson}$ computed with Eq. (16), for the control datasets including the spike-in ERCC. We find a very good agreement between these values for all the considered experiments. This shows that the variations in the number of counts per cell is the main source of the excess noise found by [2]. This also confirms that the Poisson distribution very well explains the observed variations, as already noticed with the experiment of Fig. 1. This is also confirmed by a reanalysis of these control datasets with the same strategy as in [2] but selecting cells with relatively close total number of counts per cell so decreasing the variations in the number of counts per cell [21]. The authors find values of $\phi$ close to 0.01. Another reanalysis of these datasets comparing their behaviours with simulated datasets following negative binomial of known dispersion conclude that they are not similar to simulated datasets with Poisson model but were consistent with $\phi$ values around 0.01 which makes the Poisson model sufficient in practice [22].

**Table 1:** Comparison of the values $\phi _\mathrm {fit}$ of the parameter $\phi$ found by [2] with the values $\phi _\mathrm {Poisson}$ expected from the Poisson law when taking into account the variability in the total number of counts per cell (see Eq. (16))

Experiment	$\phi _\mathrm {fit}$	$\phi _\mathrm {Poisson}$
Klein	0.0428	0.0451
Macosko	0.116	0.115
Zheng	0.0416	0.0426
Svensson1	0.0940	0.0969
Svensson2	0.369	0.379

Overall, the tests performed in this section show that the variability in droplet-based scRNA-seq is dominated by sampling noise and follows a Poisson distribution. However, it should be noted that we cannot exclude the introduction of biases at specific steps of the technique like increased or decreased reverse transcription or amplification of specific transcripts [16]. Indeed such bias would lead to a global increased or decreased presence of these genes and consequently to an overall overestimation or underestimation of each expression value. However, the variance on these genes would still follow a Poisson law.

### Estimating the distribution of expression values of a gene

We are interested here in estimating the intrinsic variability in the expression values of a given gene in a population of cells. As shown above, the output of a droplet-based scRNA-seq experiment presents not only this intrinsic variability but also an additional variability due to sampling noise, following a Poisson distribution. For lowly expressed genes and when the number of counts per cell is low, the sampling noise can actually dominate and hide the intrinsic population variability (see Additional file 1: Fig. S2).

To disentangle these two contributions, we introduce a parametric model of the intrinsic expression variability. In order to describe both high expression levels and low expression levels, the relative level of a gene is often represented in log scale or using a log transformation with a pseudo count (see Methods: GMM of the PDF for more details). While the choice of the transformation influences the result, the same strategy can be used in any transformation. We assume that the probability density function (PDF) of the transformation of the relative expression level of a gene in the population of cells can be approximated by a GMM. The number of components *m* in the mixture, as well as the Gaussians’ amplitudes *A*, means $\mu$, and widths $\sigma$, are free parameters that need to be adjusted to best reproduce the observed number of counts of the considered gene in each cell. We denote by ${\mathcal {G}}(\lambda | m, A, \mu , \sigma )$ this PDF (see Methods: Estimation of likelihood for more details). Then, for a cell *i* whose expression level for gene *g* is $\lambda _{i,g}$, the probability to get $k_{i,g}$ out of $N_i$ counts follows the Poisson distribution ${\mathcal {P}}(k_{i,g} | N_i \lambda _{i,g})$. Therefore, the likelihood ${\mathcal {L}}$ of a given set of parameters $(m,A,\mu ,\sigma )$ is written as

17 $$\begin{aligned} {\mathcal {L}}(m,A,\mu ,\sigma )&= \prod _{i=1}^n p(k_{i,g}|N_i,m,A,\mu ,\sigma )\nonumber \\&= \prod _{i=1}^n \int {\mathcal {P}}(k_{i,g}| N_i\lambda ) {\mathcal {G}}(\lambda | m,A, \mu , \sigma ) \mathrm {d}\lambda . \end{aligned}$$

We estimate this integral numerically as described in Methods: GMM of the PDF. While the set of parameters $m,A,\mu ,\sigma$ could be determined by maximizing the likelihood, we advocate here for a Bayesian approach which prevents an overfit of the data and allows to better estimate confidence intervals on the parameters and on the corresponding PDF ${\mathcal {G}}$, which is the quantity of interest in this study. We thus use a Markov chain Monte Carlo (MCMC) algorithm to explore the parameters *A*, $\mu$, $\sigma$ for a given number of components *m*. Then we combine the results obtained for different *m* by evaluating the evidence of each model using an importance sampling algorithm. These Bayesian approaches (MCMC and importance sampling algorithms) require to define priors on the model parameters. We provide more details on the algorithms and priors in Methods: Priors on parameters and MCMC algorithm. We call this algorithm baredSC for Bayesian Approach to Retrieve Expression Distribution of Single-Cell data.

**Fig. 2:**
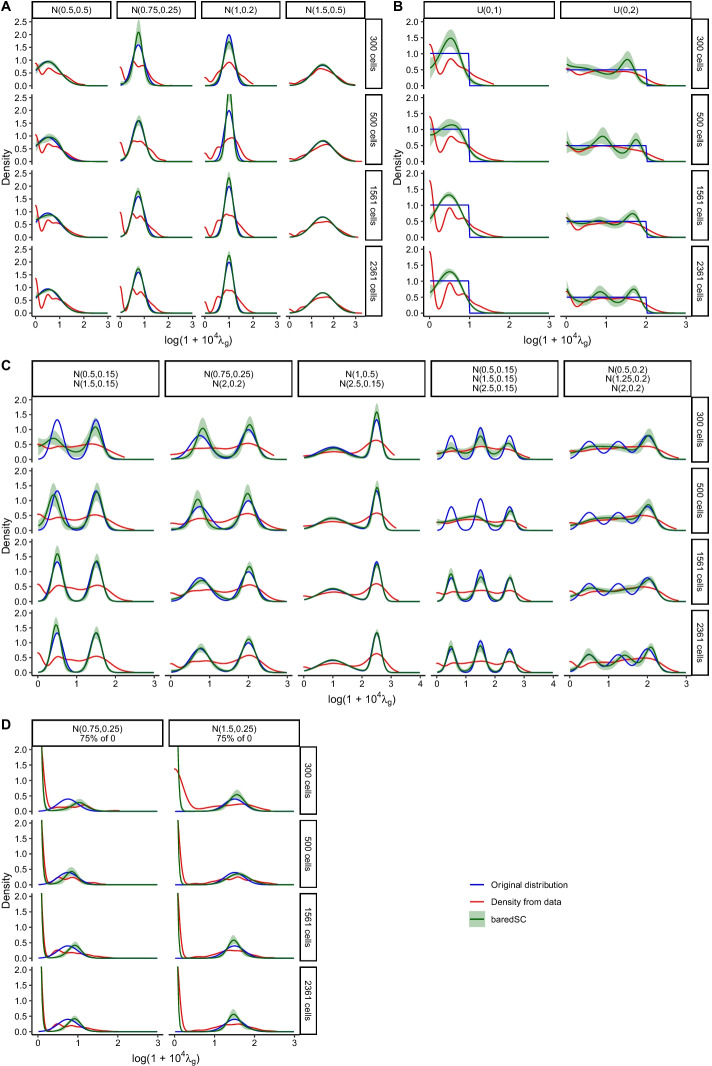
MCMC allows a good estimation of the distribution. In each simulation, the simulated distribution is plotted in blue and its characteristics are written above (N for normal, U for uniform followed by loc and scale values as in the scipy package as well as the proportion of cells with no expression in **D**). The values obtained after Poisson simulation are summarized by the red curve (Density from data). The mean PDF obtained by baredSC is depicted in green and the green area shows the quantile 16–84%. In **A**, distribution is only composed of one normal distribution, in **B**, only one uniform distribution, in **C** two or three Gaussians were used and in **D** a normal distribution for part of the cells and no expression for other

In order to test the efficiency of the algorithm to retrieve the intrinsic distribution, we simulated data using 300, 500, 1561 cells or all (2361 cells) with $N_i$ values taken from a real 10X dataset (see Methods: Simulation of data). We generated random values for the expression in each cell ($\lambda _{i,g}$) according to various intrinsic distributions on the $log(1 + 10^4 \lambda _g)$ scale: single Gaussian (Fig. 2A), uniform distribution (Fig. 2B), two or three Gaussians (Fig. 2C), or a sub-population of zeros (cells not expressing the gene) and a Gaussian (Fig. 2D). We varied the mean and the width of each of these distributions to cover a wide range of potential biological cases. Then, we simulated a scRNA-seq by randomly sampling the $k_{i,g}$ values from a Poisson law using the determined $N_i$ and $\lambda _{i,g}$ values. Finally, these simulated counts were analyzed using baredSC with the same log transformation ($log(1 + 10^4 \lambda _g)$) with up to four Gaussians in the mixture.

In the single Gaussian cases, the algorithm very efficiently approximates the intrinsic distribution, even with only 300 cells (see Fig. 2A). We plot in Fig. 2B the case of uniform distributions. While the exact shape of the original distribution cannot be exactly reproduced using a GMM, it is still reasonably well approximated. In particular, when the scale is large enough, the model uses multiple Gaussians to better reproduce a flat distribution. Such a distribution is unlikely to exists in biology, but it illustrates well the versatility of the GMM. When multi-modal distributions were simulated, baredSC could in most cases accurately identify the different modes (see Fig. 2C). The algorithm sometimes slightly deviates from the simulated distribution in the range of low expression when the number of cells is relatively low. This phenomenon is highly linked to the sparsity of the data and to the model’s degeneracy between very low expression levels. Using a higher number of cells allows to break this degeneracy. Finally, in the case of zeros plus a Gaussian, baredSC approximates the distribution with two Gaussians (see Fig. 2D). One of the Gaussians corresponds to the simulated Gaussian. The other is truncated and narrowed close to zero, such that it approximates the sub-population non-expressing cells. However, baredSC tends to predict a lower proportion of expressing cells with a higher mean expression. Again, this phenomenon is linked to the sparsity of the data and to the model’s degeneracy between very low expression and no expression. It disappears when we simulate a higher mean expression for the expressing cells (right column of Fig. 2D).

Overall, the algorithm performs very well and largely improves the estimation of the intrinsic expression distribution compared to a classical density plot (see Fig. 2).

### Comparison to Sanity

Sanity is a normalization tool aimed at correcting scRNA-seq outputs from sampling noise [15]. The Sanity model shares similar features with baredSC, as both tools use a Bayesian approach to correct from the sampling noise, which is assumed in both cases to follow a Poisson law. However, there are two main differences in the baredSC and Sanity approaches (see Additional file 1: Section 3 for more details). First, while baredSC uses a GMM (using any transformation of $\lambda _g$) to model the intrinsic expression distribution, Sanity uses a single Gaussian model expressed in $log(\lambda _g)$. We observe that baredSC’s results are similar to the ones obtained with Sanity if we restrict baredSC to use a single Gaussian in log scale (Additional file 1: Fig. S3). The Sanity model is thus simpler, which allows for the use of faster algorithms. However, it is also less flexible and precise, especially in cases where the expression distribution is multi-modal. Second, Sanity aims at correcting the counts of each cell from the Poisson noise, while baredSC focuses on uncovering the underlying intrinsic expression distribution (PDF) of a gene. Of note, while the estimation of corrected counts is not the goal of baredSC, this information can be easily computed from the inferred PDF (see Methods: Posterior per cell). The expression PDF can be estimated from Sanity’s outputs by either using the single Gaussian used to model the intrinsic expression distribution, or computing a KDE of the corrected counts, or by computing the posterior distribution from these counts and their error bars (see Additional file 1: S3.7 and Fig. S23 of [15]). However, the two last methods are less accurate than the PDF infered by baredSC when multiple Gaussians are used and can degrade the resolution of the PDF (see Additional file 1: Fig. S3).

**Fig. 3:**
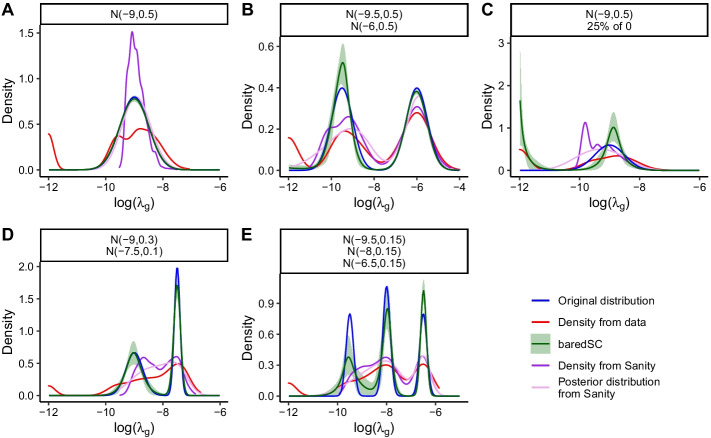
baredSC better estimates the PDF compared to Sanity when the distribution is multi-modal. In each simulation, the simulated distribution is plotted in blue and its characteristics are written above (N for normal followed by mean and scale values in log as well as the proportion of cells with no expression). The values obtained after Poisson simulation are summarized by the red curve (Density from data). The mean PDF obtained using baredSC is depicted in green and the green area shows the quantiles at 16–84%. The values obtained using Sanity are summarized by the purple (Density from Sanity) and pink (Posterior distribution from Sanity) curves

In order to compare baredSC and Sanity, we generated data using 2361 cells with the same $N_{i}$ values as for Fig. 2. We generated $\lambda _{i,g}$ values using a single Gaussian, two Gaussians, a single Gaussian in combination with a proportion of cells with no expression, or three Gaussians. In order to more easily compare the results from Sanity and baredSC, the Gaussians were defined in log scale ($log(\lambda _g)$) and we run baredSC using the same log scale. The results are displayed in Fig. 3. For the representation of the normalized counts, the expression of cells with no expression was artificially put to the minimal value. In Fig. 3A where a single Gaussian was simulated, baredSC and the posterior distribution from Sanity overlay with the distribution used to simulate the data. The density from Sanity underestimates the low values and overestimates the mean values. In Fig. 3B, we used a bimodal distribution closed to the one used in the Fig. S23 of [15]. In this case, the posterior distribution from Sanity shows a bi-modal shape, however, while the second Gaussian’s characteristics are well estimated, the first one has a larger scale than expected. Conversely, baredSC finds the characteristics of both Gaussians. In Fig. 3C, where a proportion of cells with no expression was added, baredSC identifies the two sub-populations and gives an inferred PDF very close to the generated one. The posterior distribution from Sanity is composed of a single broad Gaussian missing the bimodality (Gaussian and non-expressing cells). In Fig. 3D, where two Gaussians were used with averages smaller than -7.5, the posterior distribution from Sanity is close to a single Gaussian while baredSC results is close to the expected PDF. Finally, in the last simulation (Fig. 3E) where three Gaussians were used, the posterior distribution from Sanity is very close to the line obtained with normalized counts (Density from data) except that it corrects for cases where there was no detection. This is much less accurate that the PDF provided by baredSC. Overall, in these simulations, baredSC better estimates the distribution compared to Sanity when the distribution is produced by more than a single Gaussian. This can be explained by two factors. First, the simplified single-Gaussian model used by Sanity favors unimodal outputs. Second, for each cell, the posterior distribution of Sanity is approximated by a Gaussian, while this distribution is actually the product of the Poisson distribution with the assumed intrinsic distribution. Even for a Gaussian intrinsic distribution, as assumed in the Sanity model, this product might significantly depart from a Gaussian distribution (asymmetry and/or bimodality). Together, these two approximations might explain why the posterior distribution from Sanity is less accurate than baredSC results in multi-modal distributions.

These results demonstrate that while Sanity offers a fast solution to correct scRNA-seq outputs from sampling noise, baredSC provides much more accurate results, at the cost of computing time. Both approaches are complementary and have different scopes. The efficiency of the Sanity algorithm allows to apply it massively on all genes. This is especially useful to correct scRNA-seq from sampling noise before applying a clustering and/or a projection algorithm. Such an application would be very intensive in computer time for baredSC, which is dedicated to more in-depth studies of specific genes.

### Benchmark of baredSC computational needs

**Fig. 4:**
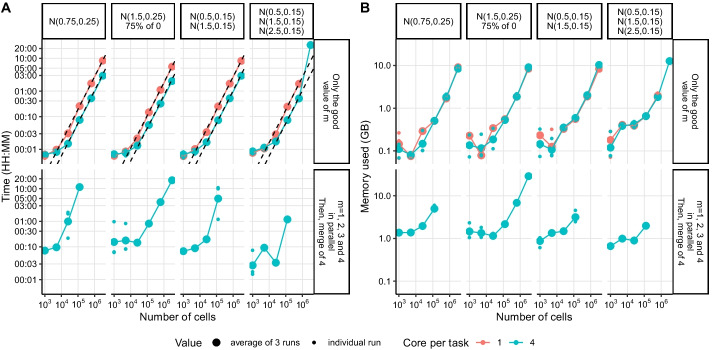
baredSC computational needs. For each run of baredSC, the computational time (**A**) and the memory used (**B**) is plotted as function of the number of cells in the input. On top panel are presented the results of run where baredSC has been run with only one value of *m* (number of Gaussians) corresponding to the expected result until convergence. On the bottom panel are presented the results of run where baredSC has been run in parallel for 1, 2, 3 and 4 Gaussians followed by a scripts which combine all 4 intermediate results

As baredSC uses a MCMC algorithm to assess the probability distribution of the parameters of the model, the computational cost of the algorithm is non-negligible. At each step of the MCMC, the cost is driven by the evaluation of the likelihood which is proportional of the number of cells *n* (see more details in Methods: GMM of the PDF). By default baredSC runs 100,000 MCMC steps, which requires to evaluate the likelihood 100,000 times. If the MCMC did not converge, we automatically rerun baredSC with 10 times more steps until the convergence is reached (see Methods). In order to get an evaluation of the time and memory needed to run baredSC as function of the number of cell, we simulated some distributions used in Fig. 2 for 1000, 5000, 25,000, 125,000, 625,000, and 3,125,000 cells (Fig. 4).

We first ran baredSC using the number of Gaussians (*m*) corresponding to the simulated data, i.e. one Gaussian for the first simulation, two Gaussians for the two next simulations and three Gaussians for the last simulation. We ran baredSC on the high performence computing server of EPFL using either one core or four cores as baredSC uses numpy which can use multiple cores. baredSC was run three times with three different seeds to take into account the variability of the node performance and the MCMC convergence. The results for the computation time is presented in Fig. 4A top panel and the memory is presented in Fig. 4B top panel. Only conditions where the convergence could be achieved in the three different seeds within 30 h are displayed. In all cases except one, the MCMC converged with 100,000 steps (see Additional file 1: Fig. S4). We can see that the computation time is indeed linear as function of the number of cells (dotted line) except in the last simulated distribution where the linearity is broken with three million cells due to convergence issues. The use of four cores is interesting as soon as the number of cells is above 25,000. For this number of cells, it takes between 2 and 4 min independently of the complexity of the distribution with one core and around 1 min and 30 s with four cores. BaredSC requires relatively low memory, around 0.3GB for 25,000 cells.

However, in the case of real datasets, the number of Gaussians (*m*) is unknown and is a parameter of the model so we need to run baredSC with any possible number of Gaussians. We decided to use one to four Gaussians which enable to cover a large variety of distributions. Then, a script uses the four intermediate results and importance sampling to give the final result. To optimize the computational run time, we ran baredSC in parallel on one to four Gaussians with four cores per task. This strategy is the one which has been used for all the figures of the paper. While with the first performance tests, the MCMC was nearly always converging at 100,000 steps, when different number of Gaussians are used, the convergence may require 1,000,000 steps or even 10,000,000 steps for a number of Gaussians which does not correspond to the simulated number of Gaussians (Additional file 1: Fig. S4). This strongly impacts the computational time (Fig 4A bottom panel). On average, the full baredSC with one to four Gaussians requires 28 min for 25,000 cells and 1.5 GB.

These computational times are not compatible with a usage of baredSC for hundreds or thousands of genes. However, it stays reasonable when the PDF needs to be precisely known for few genes in a whole dataset or in specific clusters.

### Estimating the joint distribution of two genes

As shown above, the baredSC approach is able to efficiently recover the expression distribution of a single gene. The same approach can actually be applied to two genes simultaneously to infer the expression distribution in two dimensions (2D). This is of great interest since the study of pairwise correlations between genes can help to better understand gene regulatory networks. Similarly to the case of a single gene, we assume that the distribution can be approximated by a GMM. In the 2D case, each Gaussian in the GMM depends on six parameters: the amplitude, the mean of x and y, the scale of x and y and the correlation. The detailed equations for the 2D application of baredSC can be found in Methods: baredSC in 2D. The same Bayesian approach (MCMC and importance sampling) as in the 1D case is used to explore the parameters. In addition to the parameters and the corresponding expression distribution in 2D, one can deduce from the MCMC posteriors a confidence interval for the Pearson correlation between the expression of the two genes, as well as a one-sided *p* value (see Methods: Estimation of the p value for the correlation).

**Fig. 5:**
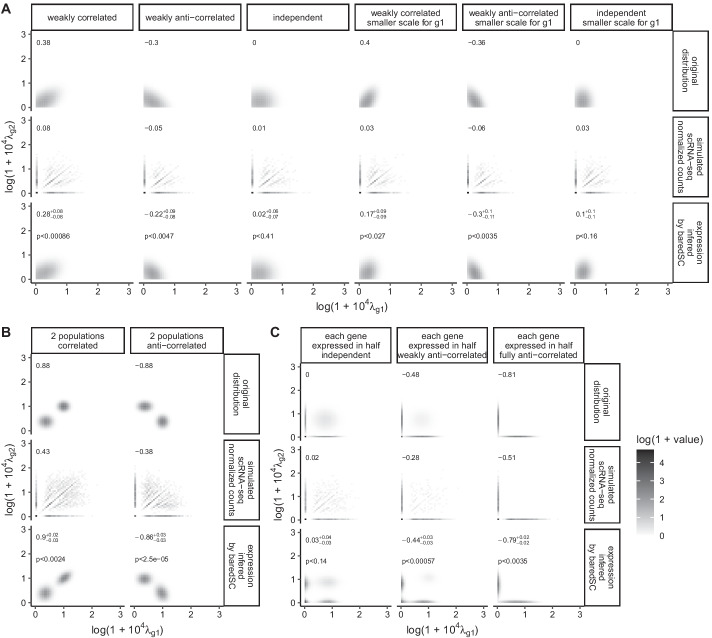
baredSC allows a good estimation of 2D expression distributions. In each simulation, the PDF of the original distribution is plotted in the top panel. The values obtained after Poisson simulation are plotted in the middle panel. The mean PDF obtained by baredSC is plotted in the bottom. In the top left corner is written the Pearson’s correlation coefficient and for baredSC its confidence interval. For baredSC, an estimation of the one-sided *p* value is displayed. In **A**, distribution is only composed of one truncated normal distribution, in **B**, 2 normal distributions are used, and in **C** each gene is expressed with a Gaussian distribution in half of the cells

We simulated data to test the accuracy of baredSC in 2D. We used the same number of cells and the same $N_{i}$ values as in the 1D case. We generated random values for the expression of both genes x and y in each cell ($\lambda _{i, g1}$ and $\lambda _{i, g2}$) using various distributions on the $log(1 + 10^4\lambda _g)$ scale described below. Then, we sampled $k_{i, g1}$ and $k_{i, g2}$ using Poisson law with parameters $\lambda _{i, g1}N_i$ and $\lambda _{i, g2}N_i$ respectively. These simulated counts were analyzed using baredSC with the same log transformation ($log(1 + 10^4 \lambda _g)$) with one to four Gaussians. The results obtained using all cells are presented in Fig. 5, while the results obtained on smaller subgroups are presented in Additional file 1: Fig. S5. For each generated dataset, we plot in Fig. 5 the distribution used to generate the intrinsic expression values (original distribution), the distribution of normalized simulated raw counts ($k_{i,g}/N_i$, simulated scRNA-seq normalized counts), and the distribution inferred with our approach (baredSC).

**Fig. 6:**
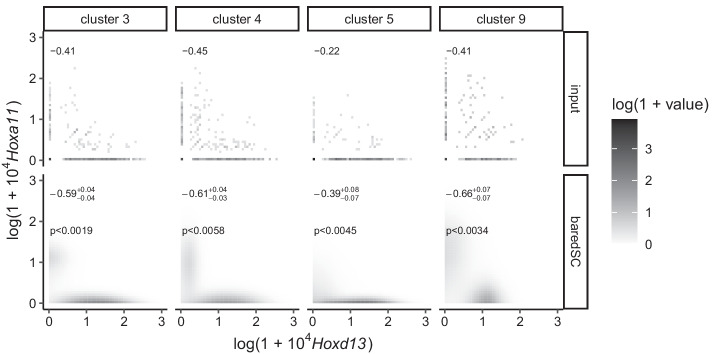
baredSC retrieves the anti-correlation between *Hoxa11* and *Hoxd13* in embryonic distal limb scRNA-seq. Heatmaps showing the PDF of normalized expression of *Hoxa11* and *Hoxd13*. Top panels provide the PDF from input data while bottom panels show the result of baredSC. In the top left corner is written the Pearson’s correlation coefficient and for baredSC its confidence interval. For baredSC, an estimation of the one-sided *p* value is displayed

We first generated the data using distributions composed of a single truncated 2D Gaussian (Fig. 5A). In the three first columns, the mean of *g*1 and *g*2 and the scale of *g*1 and *g*2 are all equal to 0.25. The correlation is set to 0.5 (weakly correlated), − 0.5 (weakly anti-correlated) and 0 (independent). We can see that the PDF of the normalized data (simulated scRNA-seq normalized counts) is very noisy and a lot of signal goes on the axes (i.e., one or both genes are not detected). The corresponding Pearson’s correlation coefficient (top left of plot) is close to 0 in the three cases while one would expect values around $\pm \,0.3$ for the weakly (anti-)correlated cases, in the absence of sampling noise (generated expression). The mean PDF recovered by baredSC (bottom row) is very similar to the original PDF (top row) in these three cases. The Pearson’s correlation coefficient estimated from the MCMC posteriors (top left of each plot in bottom row) is compatible with the value computed from the original PDF (within confidence interval). Moreover, the estimation of the one-sided *p* value is significant only in cases where the correlation of the original distribution was non-zero, as expected.

In the three last columns of Fig. 5A, the simulated Gaussians are identical to the first three except that the scale of the gene *g*1 is 0.15 instead of 0.25. Despite the fact that the smaller scale increases the number of drop-out events, baredSC results are still very close to the input PDF.

In Fig. 5B, we split the cells in two equally sized populations. Each population expresses both genes following a 2D Gaussian without correlation. The means of both 2D Gaussians were chosen in order to introduce a correlation (first column of Fig. 5B) or an anti-correlation (second column). The correlation or anti-correlation is already perceptible in the PDF of the normalized counts (simulated scRNA-seq normalized counts). However, the presence of two populations is totally hidden by the sampling noise. The results of baredSC (bottom row) highlight the two distinct populations. The correlation coefficient is very well approximated, especially when compared to the one evaluated on the raw data.

Finally, we generated three different distributions where each of the two genes is expressed in only half of the cells (Fig. 5C). In the first column (each gene expressed in half independent), the cells which do not express the gene *g*1 or the gene *g*2 were chosen independently. In the second column, the two genes are partially anti-correlated. Both genes are expressed in 10% of the cells, neither is expressed in 10%, and gene *g*1 (resp. *g*2) is expressed while gene *g*2 (resp. *g*1) is not in 40%. In the third column, each cell is expressing either *g*1 or *g*2 (each gene expressed in half fully anti-correlated). The estimated correlation coefficients are compatible with the ones obtained on the simulated PDF (generated expression). The shape of the PDF also resembles what was expected. We can however notice that, similarly to what was observed in Fig. 2d for the 1D case, the means of the expressed populations of cells are slightly over estimated. Once again, this can be explained by the sparsity of the data and by the model’s degeneracy.

While it is visually very difficult to interpret the 2D PDF of normalized counts (simulated scRNA-seq normalized counts) for lowly expressed genes, baredSC provides a good approximation of the shape of the original PDF. Similarly to what has been observed in one dimension, when baredSC is compared to Sanity, we observe that except in the single Gaussian without any correlation, baredSC outperform Sanity (Additional file 1: Fig. S6).

### Applications to real datasets

In order to illustrate the power of baredSC and its potential use, we apply it to two datasets.

#### *Hoxd13*-*Hoxa11* anti-correlation in embryonic distal limb

HoxA and HoxD genes are key transcription factors involved in limb patterning [23]. Their expression domain is tightly regulated in space and time. When the limb grows, *Hoxa11* expression has been described as restricted to the proximal domain which will become the arm while *Hoxd13* is expressed in the distal domain which will become the digits. [24–26] showed that *Hoxd13* represses the transcription of *Hoxa11* in the embryonic distal limb leading to two distinct domains of expression. We use the scRNA-seq from [27] which has been generated from embryonic forelimbs. A clustering analysis revealed 17 clusters. Using *Hoxd13* as a marker, 4 clusters (3, 4, 5, 9) were attributed to the distal part of the limb (see [27]). Unexpectedly, *Hoxa11* was not totally absent from these clusters. We run baredSC on each of these 4 clusters allowing one to four Gaussians for *Hoxd13* and *Hoxa11* (Fig. 6). In all four clusters, we clearly see a depletion of cells with simultaneous high expression of both genes. As a consequence, the correlation coefficient is significantly negative in all four clusters. These results are thus in agreement with the literature.

#### Multi-modal expression of *Pitx1* in embryonic hindlimb

The gene *Pitx1* encodes for a transcription factor expressed in the embryonic hindlimb. It is responsible for the leg patterning. This gene is not expressed in the forelimb and a gain of expression in this domain induces an arm-to-leg phenotype [28, 29]. Its expression is controlled by many enhancers, one of them is *Pen* which accounts for 30–50% of the expression [29, 30]. Rouco et al. [30] used scRNA-seq and flow cytometry to investigate whether *Pitx1* was homogeneously expressed across hindlimb cells. They also studied the changes induced by the deletion of the *Pen* enhancer. While scRNA-seq directly measures the level of expression of *Pitx1*, the flow cytometry data measures the level of fluorescence thanks to a GFP-sensor introduced in close proximity to the *Pitx1* promoter. The measure of fluorescence of each cell by flow cytometry should be highly correlated to the level of mRNA of *Pitx1*. However, the exact relationship between the mRNA level and the fluorescence is not known.

**Fig. 7:**
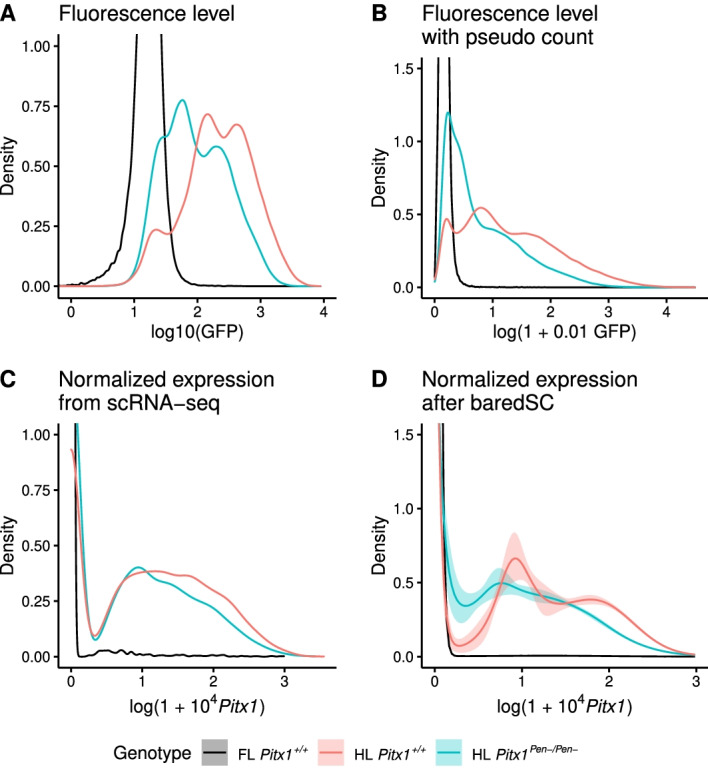
baredSC retrieves the trimodal expression of *Pitx1* in embryonic limb scRNA-seq. **A** Distribution of fluorescence intensity obtained by flow cytometry in regular log scale. **B** Distribution of fluorescence intensity on log scale with pseudo count to be closer to the Seurat scale. **C** Distribution of normalized expression from scRNA-seq as provided by the Seurat package. **D** Distribution of normalized expression from scRNA-seq as provided by baredSC

In this study, there are three conditions, the forelimb (FL) wild-type (*Pitx1*$^{\mathrm {\textit{+/+}}}$) which has no expression of *Pitx1*, the hindlimb (HL) wild-type which is considered as a tissue expressing *Pitx1* and the HL mutant where the *Pen* enhancer has been deleted (*Pitx1*$^{\mathrm {\textit{Pen-/Pen-}}}$). Both flow cytometry and scRNA-seq data experiments were produced for these 3 samples. We propose a reanalysis of these data. The flow cytometry data are usually represented in log scale (Fig. 7A). It shows the presence of high level of background in absence of expression. Indeed, in the FL *Pitx1*$^{\mathrm {\textit{+/+}}}$ sample in black, the fluorescence level has a relatively wide scale. Rouco et al. [30] show that the expression of *Pitx1* in the HL wild-type (red curve) is trimodal (Figs. 7A and 3A of [30]). The first mode is included in the range of the FL *Pitx1*$^{\mathrm {\textit{+/+}}}$ so it corresponds to cells which do not express *Pitx1*. Expressing cells can be divided into highly-expressing cells and lowly-expressing cells. The sample HL *Pitx1*$^{\mathrm {\textit{Pen-/Pen-}}}$ (blue curve) also exhibits a trimodal distribution but the average expression in low expressing and high expressing cells is decreased compared to the wild-type condition and there is an increased proportion of non expressing cells. These data are consistent with the expected decrease of expression by 30-50%. In order to compare more easily the flow cytometry data with the scRNA-seq expressed in $\log (1 + 10^4 X_i)$, we transformed the fluorescence in $\log (1 + 0.01 F_i)$ (Fig. 7B). This transformation still highlights the trimodal expression of the HL *Pitx1*$^{\mathrm {\textit{+/+}}}$. When the scRNA-seq results are displayed as density plots (Fig. 7C or Fig. 4A of [30]), one cannot distinguish the three modes in the HL *Pitx1*$^{\mathrm {\textit{+/+}}}$. However, classical density plots are very sensitive to sampling noise, and do not allow a precise determination of the expression distribution. Applying baredSC on this dataset enables to a better characterization of the shape of the expression distribution (Fig. 7D). The three modes are uncovered: a first one with non-expressing cells approximated by a truncated Gaussian along the y axis, a second one with a mean value around 1, and a third one with a mean value around 2. Because of the background in flow cytometry and the degeneracy of scRNA-seq at very low expression, the comparison of the PDFs from flow cytometry (Fig. 7B) and scRNA-seq (Fig. 7D) on the left part of the plots is difficult. However, at higher expression levels (right part), the PDF are very similar. We also run baredSC in regular log scale (Additional file 1: Fig. S7) and confirm this high similarity with the flow cytometry PDF. These results show that the trimodal expression identified by flow cytometry is indeed present in the scRNA-seq. It also demonstrates how baredSC can improve our description of expression variability within a scRNA-seq from a complex tissue.

## Discussion

### Poisson distribution is sufficient to model droplet-based scRNA-seq

In this study, we show that most of the technical variability is well approximated by the Poisson distribution. While other papers have justified the use of the Poisson distribution only on a theoretical ground [15, 31], we base our conclusions on the analysis of control scRNA-seq. We compare estimators of the mean and variance of the scaled expression of each gene in each control dataset, and show that for most genes the two estimators (mean and normalized variance) are close to each other, which is a characteristic of the Poisson distribution. Using the same datasets but with non-scaled data, [2] concluded that the negative binomial, which models an additional variability compared to Poisson, was the best fitting law. However, we show that this excess of variance is simply due to the variability in the total number of reads per cell, and confirm that the Poisson law is well suited to model droplet-based scRNA-seq. Our conclusions are in line with recently published reanalyses of these control datasets [21, 22].

We note that our demonstration is limited to droplet-based scRNA-seq as we only found control datasets available for these techniques. Similar control datasets for other scRNA-seq techniques would be very valuable to assess their technical variability and build well justified mathematical models.

### baredSC performances

The distribution of expression of a gene from scRNA-seq data is often represented using density/violin plots. This representation mixes biological variation and variation coming from the technique (mainly sampling). baredSC is designed to disentangle these two effects, and retrieve the biological distribution of expression. Using simulated data we show that baredSC indeed outperforms classical representations. It allows to retrieve precisely multi-modal expression distribution even when they are not distinguishable in the input data due to sampling noise. In these cases, baredSC results are also more accurate than the posterior distribution from Sanity, another recently published tool which aims at denoising scRNA-seq data. The 2D version of baredSC also allows a better evaluation of the correlation between genes than classical methods and Sanity, and provides an accurate PDF even for lowly expressed genes.

However, we observed some conditions where the inferred distribution deviates from the generated distribution. First, while we have demonstrated the versatility of the GMM to approximate different types of distributions (e.g. uniform distribution), the fit cannot be perfect for distributions that are not Gaussian mixtures. We note that the baredSC approach could easily be adapted to use another family of distributions, but the GMM seems a reasonable approximation for real applications. Second, when the population of cells is split into a group that does not express the considered gene and a group that expresses it at low level, the inferred distribution tends to overestimate the non-expressing group and overestimate the mean expression level in the expressing group. This phenomenon is highly linked with the degeneracy of the model. In 2D, as the model is more complex, the number of cells can be a limitation to accurately estimate the PDF when the expression level is low.

As baredSC uses a MCMC algorithm to infer the model parameters, the computational cost of the algorithm is non-negligible. It takes typically 10 min with four times four cores to run one to four Gaussians, for each gene of the examples of Fig. 2 using all 2361 cells. A quicker alternative would be the frequentist approach which maximizes the likelihood. In this particular case, this method is error prone as there is a risk of overfit and a risk to find parameters which corresponds to a local maximum of likelihood which does not represent the global maximum. Indeed, the high degeneracy of the problem is a big challenge with this approach. In addition, the evaluation of error bars is much more difficult with a frequentist approach than with a MCMC approach. This is why we use a MCMC algorithm to get a confidence interval on the inferred PDF.

### baredSC applications

In the single gene use case, baredSC could replace the widely used violin plots to describe the expression of few genes of high interest across different conditions or clusters. Indeed, baredSC allows to retrieve very precisely the expression distribution of genes, even in multi-modal cases and with strong sampling noise. This accuracy comes at the cost of computation time. Moreover, baredSC requires some manual checks, such as the convergence of the MCMC, and the fact that the range of considered expression levels were correctly chosen. For these reasons, this tool is more appropriate to in-depth analysis of specific genes than large-scale applications. As shown in the examples, it can be used both on a whole single-cell dataset or on cells belonging to specific clusters. Potential applications for a single gene are modality description or estimation of proportion of cells expressing a given gene, as well as comparisons between samples or clusters.

We apply it to a real biological scRNA-seq dataset to study the modality of the expression of *Pitx1*. We demonstrate that the fluorescence measurement of the GFP sensor, integrated in close proximity of *Pitx1* promoter, is highly correlated with the mRNA level of the gene. The inference of PDF in 2D is of great interest for the study of genes’ interactions or its comparison between clusters of cells. We apply it to confirm the anti-correlation between *Hoxa11* and *Hoxd13* in the developing limb.

## Conclusions

scRNA-seq data usually present a high level of sparsity. The use of normalized counts to describe variability among a cell population mixes intrinsic variability and variability coming from the sampling noise. baredSC allows to retrieve the intrinsic variability providing the inferred expression distribution either for a single gene or for two genes simultaneously. As such, baredSC represents a valuable tool to perform in-depth analysis on few genes of interest.

## Availability and requirements

Project name: baredSC
Project home page: https://github.com/lldelisle/baredSC
Operating system(s): Linux and MacOS (both as python package and standalone scripts), Windows (only as python package)
Programming language: python
Other requirements: python $\ge$ 3.7, numpy, matplotlib, pandas, scipy, samsam
License: GNU General Public License v3.0

## Methods

### Comparison between the Negative Binomial and the Poisson distribution

In Fig. 1, the coefficient of the Negative Binomial was fitted using the nls (Nonlinear Least Squares) function of R on the formula: $log({\tilde{V}}_g) \sim log(M_g + a M_g^2)$ starting with $a=0$.

### GMM of the PDF

In the scRNA-seq field, the most popular analysis packages (Seurat and Scanpy) uses a log transformation of the normalized counts to be able to see both highly expressing cells and lowly expressing cells. To handle the numerous cases where the gene was not detected, they use a transformation of type $log(1 + N_{t} X_{i,g})$ where $N_{t}$ is the targeted number of counts used to normalize $X_{i,g}$ values. In the Seurat package $N_{t}$ is fixed to $10^4$ while in Scanpy it is set by default to the median of $N_{i}$ or it can be user defined. However, the pseudo-count in log may introduce a distortion and users may prefer to work in regular log scale. In baredSC, both scales have been implemented, and the PDF is modeled by a fixed number of Gaussians in the chosen scale. We define

18 $$\begin{aligned} x_g = \log (1 + N_t\lambda _{g}), \end{aligned}$$

in the case of the Seurat/Scanpy scale or

19 $$\begin{aligned} x_g = \log (\lambda _{g}), \end{aligned}$$

in the regular log scale case. We denote by *l* the mapping between *x* and $\lambda$: $x = l(\lambda )$ and $\lambda = l^{-1}(x)$. In practice, we work on a finite interval $[\lambda _\mathrm {min},\lambda _\mathrm {max}]$ of expression levels, corresponding to $[x_\mathrm {min},x_\mathrm {max}]$ for the transformed expression *x*, and model the PDF using a mixture of truncated Gaussians.

The likelihood is:

20 $$\begin{aligned} {\mathcal {L}}(\theta )&= \prod _{i=1}^n p(k_{i,g}|N_i,\theta ) \end{aligned}$$

21 $$\begin{aligned}{\mathcal {L}}(\theta )&= \prod _{i=1}^n \int _{\lambda _\mathrm {min}}^{\lambda _\mathrm {max}} {\mathcal {P}}(k_{i,g}| N_i\lambda ) {\mathcal {G}}(\lambda | \theta ) \mathrm {d}\lambda \nonumber \\&= \prod _{i=1}^n \int _{x_\mathrm {min}}^{x_\mathrm {max}} {\mathcal {P}}(k_{i,g}| N_i l^{-1}(x)) G(x | \theta ) \mathrm {d}x , \end{aligned}$$

where $\theta = (m,A,\mu ,\sigma )$ is the set of model parameters, and

22 $$\begin{aligned} G(x | \theta )&= \sum _{j=1}^m a_{j} \exp \left( -\frac{(x-\mu _j) ^2}{2\sigma _j^2}\right) ,\nonumber \\ a_j&= A_j \left( \int _{x_\mathrm {min}}^{x_\mathrm {max}} \exp \left( -\frac{(x-\mu _j) ^2}{2\sigma _j^2}\right) \mathrm {d}x\right) ^{-1},\nonumber \\ {\mathcal {G}}(\lambda | \theta )&= G(l(\lambda ) | \theta ) l'(\lambda ). \end{aligned}$$

### Estimation of likelihood

Unfortunately, the integral in Eq. (21) cannot be computed analytically, and we thus evaluate it numerically using a Riemann sum

23 $$\begin{aligned} L_i(\theta )&= \int _{x_\mathrm {min}}^{x_\mathrm {max}} {\mathcal {P}}(k_{i,g}| N_i l^{-1}(x)) G(x | \theta ) \mathrm {d}x\nonumber \\&\approx \sum _{q=1}^{n_x} {\mathcal {P}}(k_{i,g}| N_i l^{-1}(x_q)) G(x_q | \theta ) \delta x, \end{aligned}$$

where $x_q$ are the centers of the $n_x$ equally sized bins, and $\delta x = (x_\mathrm {max}-x_\mathrm {min})/n_x$ is the width of each bin. In this integral, the Poisson part (${\mathcal {P}}(k_{i,g}| N_i l^{-1}(x))$) does not depend on the model parameters. It can thus be pre-computed on a given grid of *x* values for each cell, and stored in a $n\times n_x$ matrix *P*. On the contrary, the GMM part ($G(x | \theta )$) depends on the model parameters but is the same for all cells. Therefore, for a given set of parameters, it must be computed on the grid of *x* only once for all cells, and stored in a vector $\gamma (\theta )$ of size $n_x$. We thus obtain the vector *L* as the dot product

24 $$\begin{aligned} L(\theta ) \approx P \gamma (\theta ). \end{aligned}$$

Then the likelihood is simply given by

25 $$\begin{aligned} {\mathcal {L}}(\theta ) = \prod _{i=1}^n L_i(\theta ). \end{aligned}$$

The computational cost of this method is dominated by the dot product of Eq. (24). Indeed, the matrix *P* can be pre-computed so its cost is negligible, the cost of computing the vector $\gamma$ scales as ${\mathcal {O}}\left( n_x\right)$, while the cost of the dot product scales as ${\mathcal {O}}\left( n n_x\right)$. Therefore, a trade-off between resolution and cost must be found to choose the number of bins $n_x$.

However, since the cost of computing *P* and $\gamma$ is much lower than the cost of the dot product, *P* and $\gamma$ can actually be computed on a finer grid to improve precision. We thus subdivide each of the $n_x$ original bins into $n_s$ sub-bins and compute the average value of ${\mathcal {P}}$ over these sub-bins. Similarly, each of the $n_x$ original bins were divided into $n_r$ sub-bins and compute the average value of *G* over these sub-bins.

26 $$\begin{aligned} P_{i,q}&= \frac{1}{n_s}\sum _{s=1}^{n_s} {\mathcal {P}}(k_{i,g}| N_i l^{-1}(x_{q,s})),\nonumber \\ \gamma _{q}(\theta )&= \frac{1}{n_r}\sum _{r=1}^{n_r} G(x_{q,r}|\theta ). \end{aligned}$$

### Priors on parameters

We chose the priors on the GMM parameters as follows

- Number of Gaussians *m*: uniform distribution over $[\![1, m_\mathrm {max}]\!]$ (with $m_\mathrm {max}=4$ in this article);
- Amplitudes $A_j$: uniform distribution over [0, 1], with the additional condition $\sum _{j=1}^m A_j = 1$;
- Means $\mu _j$: uniform distribution over $[x_\mathrm {min}-3\sigma _j, x_\mathrm {max} + 3\sigma _j]$;
- Scales $\sigma _j$: log-uniform distribution over $[\sigma _\mathrm {min}, x_\mathrm {max}-x_\mathrm {min}]$ (with $\sigma _\mathrm {min}=0.1$ in this article).

This model is symmetric in the sense that two Gaussians can be arbitrarily swapped without changing the results. This degeneracy can deteriorate the convergence of the MCMC algorithm. We thus prevent any swapping to ensure the uniqueness of the solution.

### MCMC algorithm

We use the samsam python package (https://gitlab.unige.ch/Jean-Baptiste.Delisle/samsam) which is a scaled adaptive Metropolis algorithm [32–34]. We first apply a burning phase with simulated annealing in order to prevent the MCMC from being trapped in local minima. After this burning phase, the proper MCMC is run to sample the posterior distribution. The convergence can be evaluated using the auto-correlation function of the parameters. One can extract from this measure, the effective number of samples.

### Posterior per cell

Once the MCMC has converged, we evaluate the PDF $G^*(x)$ as the average of all PDF obtained at each sample of the MCMC. This PDF can be used to retrieve the posterior distribution of expression for each cell:

27 $$\begin{aligned} p(x|k_i,N_i) = \frac{{\mathcal {P}}(k_{i,g}| N_i l^{-1}(x)) G^*(x)}{\int _{x_\mathrm {min}}^{x_\mathrm {max}} {\mathcal {P}}(k_{i,g}| N_i l^{-1}(x)) G^*(x) dx} \end{aligned}$$

Using this we can get the expected value of $x_i$:

28 $$\begin{aligned} {{\,\mathrm{{\mathbb {E}}}\,}}(x_i) = \int _{x_\mathrm {min}}^{x_\mathrm {max}} p(x|k_i,N_i) x \mathrm {d}x \end{aligned}$$

That we can evaluate numerically using a Riemann sum. Similarly we can get the variance:

29 $$\begin{aligned} {{\,\mathrm{var}\,}}(x_i)&= {{\,\mathrm{{\mathbb {E}}}\,}}(x_i^2) - ({{\,\mathrm{{\mathbb {E}}}\,}}(x_i))^2 \end{aligned}$$

30 $$\begin{aligned} {{\,\mathrm{var}\,}}(x_i)&= \int _{x_\mathrm {min}}^{x_\mathrm {max}} p(x|k_i,N_i) x^2 \mathrm {d}x - \left( \int _{x_\mathrm {min}}^{x_\mathrm {max}} p(x|k_i,N_i) x \mathrm {d}x\right) ^2 \end{aligned}$$

### baredSC in 2D

The extension of baredSC to the 2D case is very similar to the 1D case. We use either the “Seurat” scale (Eq. (18)) or the regular log scale (Eq. (19)) and define $x_1 = l(\lambda _{g_1})$, $x_2 = l(\lambda _{g_2})$ for two genes $g_1$ and $g_2$, where *l* is the chosen scale mapping. Each Gaussian of the GMM is defined by 6 parameters: the amplitude ($A_j$), the means of $x_1$ and $x_2$ ($\mu _{1,j}$ and $\mu _{2,j}$), their scales ($\sigma _{1,j}$ and $\sigma _{2,j}$), and the correlation ($\rho _j$). The likelihood is written as

31 $$\begin{aligned} {\mathcal {L}}(\Theta )&= \prod _{i=1}^n p(k_{i,g}|N_i,\Theta )\nonumber \\&= \prod _{i=1}^n \int _{x_{2,\mathrm {min}}}^{x_{2,\mathrm {max}}} \int _{x_{1,\mathrm {min}}}^{x_{1,\mathrm {max}}} {\mathcal {P}}(k_{i,g_1}| N_i l^{-1}(x_1)) {\mathcal {P}}(k_{i,g_2}| N_i l^{-1}(x_2)) G(x|\Theta ) \mathrm {d}x , \end{aligned}$$

where $\Theta = (m,A,\mu _1,\mu _2,\sigma _1,\sigma _2,\rho )$ is the set of model parameters, and

32 $$\begin{aligned} G(x | \Theta )&= \sum _{j=1}^m a_{j}\exp \left( -\frac{1}{2} (x-\mu _{.,j})^\mathrm {T} C_j^{-1} (x-\mu _{.,j})\right) ,\nonumber \\ a_j&= A_j \left( \int _{x_{2,\mathrm {min}}}^{x_{2,\mathrm {max}}} \int _{x_{1,\mathrm {min}}}^{x_{1,\mathrm {max}}} \exp \left( -\frac{1}{2} (x-\mu _{.,j})^\mathrm {T} C_j^{-1} (x-\mu _{.,j})\right) \mathrm {d}x\right) ^{-1}, \end{aligned}$$

with

33 $$\begin{aligned} C_j = \begin{pmatrix} \sigma _{1,j}^2 & \sigma _{1,j} \sigma _{2,j} \rho _j\\ \sigma _{1,j} \sigma _{2,j} \rho _j & \sigma _{2,j}^2\\ \end{pmatrix}. \end{aligned}$$

The double integral in Eq. (31) is computed numerically in a very similar manner as in the 1D case, but this time by defining bins on a 2D grid. The priors are the same as in the 1D case, and we take a prior for the correlation $\rho _j$ with a normal distribution of mean 0 and scale 0.3 truncated over $[-0.95,0.95]$ in order to limit the false positive (anti-)correlation detection.

### Estimation of the *p* value for the correlation

If the correlation between the expression of two genes is suspected to be of a given sign *s*, we define the one-sided *p* value $\alpha$ as the probability for the correlation to actually be of the opposite sign $-s$. To estimate this *p* value, we first compute the Pearson’s correlation coefficient from the 2D PDF for each sample of the MCMC. We thus obtain samples from the posterior distribution of the correlation coefficient. Let’s denote by *k* the number of independent samples for which the correlation is of sign $-s$ and *n* the total number of independent samples. We have (Bayes formula)

34 $$\begin{aligned} p(\alpha |k) = \frac{p(k|\alpha )p(\alpha )}{p(k)}, \end{aligned}$$

where $p(k|\alpha )$ can be approximated by a Poisson distribution of parameter $n\alpha$ (in the limit of low $\alpha$ and high *n*)

35 $$\begin{aligned} p(k|\alpha ) = \frac{(n\alpha )^k}{k!}e^{-n\alpha }. \end{aligned}$$

We additionally assume a uniform prior for $\alpha$. The conditional expectation of $\alpha$ knowing *k* is thus

36 $$\begin{aligned} {{\,\mathrm{{\mathbb {E}}}\,}}(\alpha |k) = \int _0^1 \alpha p(\alpha |k) \mathrm {d}\alpha = \frac{k+1}{n}\frac{I_{k+1}}{I_k}, \end{aligned}$$

with

37 $$\begin{aligned} I_k = \int _0^1 \frac{(n\alpha )^k e^{-n\alpha }}{k!} \mathrm {d}\alpha = \frac{1}{n}\left( 1-e^{-n}\sum _{j=0}^{k}\frac{n^j}{j!}\right) . \end{aligned}$$

For *n* sufficiently large, we have

38 $$\begin{aligned} I_k \approx \frac{1}{n}, \end{aligned}$$

and

39 $$\begin{aligned} {{\,\mathrm{{\mathbb {E}}}\,}}(\alpha |k) \approx \frac{k+1}{n}. \end{aligned}$$

Similarly one can show that the variance is ${{\,\mathrm{var}\,}}(\alpha |k)\approx \frac{k+1}{n^2}$. The *p* value provided on Figs. 5 and 6 of the article and Additional file 1: Figs. S4 and S5 is the 1-$\sigma$ upper limit $p < {{\,\mathrm{{\mathbb {E}}}\,}}(\alpha |k) + \sqrt{{{\,\mathrm{var}\,}}(\alpha |k)} = \frac{k+1+\sqrt{k+1}}{n}$.

### Simulation of data

In order to get realistic values for $N_{i}$, we extracted the $N_{i}$ from the cells which were attributed to NIH3T3 in the dataset provided by 10X with the same criteria used by [2]. Then, 300 cells were randomly attributed to a first group, 500 cells to another one and the others to a third one. The expressions were then generated by python scripts available at https://github.com/lldelisle/scriptsForLopezDelisleEtAl2021.

### baredSC analysis

bared v1.0.0 was run with default parameters. The number of samples, starting at 100,000 was increased by 10 times until reaching a number of effective samples above 200 (parameter --minNeff 200). However, Fig. 3 and Additional file 1: Figs. S3, S6 and S7 were generated using the --scale log option to use a regular log scale.

### Data representation

All the figures were made in R (https://www.r-project.org/) with ggplot2 [35] and ggpubr (https://rpkgs.datanovia.com/ggpubr/).

#### Single gene PDF

In Figs. 2 and 3 , where simulations were run for a single gene, the blue curve displays the PDF of the distribution that has been used to simulate the data (Original distribution). The red curve displays an estimation of the PDF of the normalized expression values obtained after simulation ($k_{i,g}/N_i$). It was obtained by using the R function ‘density’ which is widely used by R users, in particular in the Seurat violin plots. This function performs a kernel density estimation but the kernel width is automatically adjusted. The green line and area are obtained from baredSC. At each step of the MCMC, a PDF can be evaluated on a range of values ($log(1+10^4\lambda _g)$ or $log(\lambda _g)$) using the values of the parameters at this step. For each value of the range of $log(1+10^4\lambda _g)$ or $log(\lambda _g)$, the mean across all steps was calculated and plotted as a green line. Similarly, for each value the range, the 16 and 84 percentile over all MCMC steps were evaluated and plotted as a green area. In Fig. 3, the purple and pink lines were obtained from Sanity. The purple curve was obtained by using the R function ‘density’ on the inferred log transcription quotients (LTQs). The pink curve was obtained by computing the average of the posterior distribution of each cell which was approximated by a Normal distribution whose parameters are the inferred LTQs and the error value on the LTQ.

#### Two-dimension PDF

For each sample of the MCMC obtained by baredSC, the 2D PDF was computed using the parameter values on a grid of x and y (x and y corresponding to $log(1+10^4\lambda _g)$ or $log(\lambda _g)$). For each bin, the average of all PDF was computed and is displayed on the last row of Fig. 5 labelled ‘expression inferred by baredSC’. The PDF of the distribution used to simulate data was computed on the same grid and is plotted on the first row labeled ‘original distribution’. In order to represent the simulated data, each cell was assigned to a bin using the normalized expression ($k_{i,g1}/N_i$ and $k_{i,g2}/N_i$), similarly to a 2D histogram. The counts of cells were then normalized to take into account the bin size. The correlation coefficient displayed on the Fig. 5 is obtained from the binned PDF.

## Supplementary Information

**Additional file 1**. Supplementary text and figures.

## Data Availability

The datasets analyzed in Fig. 1 are reanalysis of [2] and available at https://figshare.com/projects/Zero_inflation_in_negative_control_data/61292. The scRNA-seq datasets analysed in Fig. 6 and Fig. 7 are available on GEO with accessible numbers GSE165495 and GSE168632 respectively. baredSC is a python open-source package hosted on github at https://github.com/lldelisle/baredSC and can be installed by pip via PyPI or by conda via bioconda. For this paper, version 1.0.0 (http://doi.org/10.5281/zenodo.4897182) was used. All the scripts needed to reproduce all the figures of the paper are available at https://github.com/lldelisle/scriptsForLopezDelisleEtAl2021.
