# Supplementary material for: baredSC: Bayesian approach to retrieve expression distribution of single-cell data
Source: BMC Bioinformatics. 2022 Jan 12;23:36. doi: 10.1186/s12859-021-04507-8 (PMC8756634; doi:10.1186/s12859-021-04507-8)
Supplement: Supplementary file 1 — Additional file 1. Supplementary text and figures. [file 12859_2021_4507_MOESM1_ESM.pdf]

Supplementary Text and Figures for **baredSC: Bayesian Approach to Retrieve Expression Distribution of Single-Cell data**

Lucille Lopez-Delisle, Jean-Baptiste Delisle

December 8, 2021

1 ERCC show an increased variance

When scRNA-seq experiments are performed, some manufacturers recommend to spike-in ERCC (External RNA Control Consortium). These are synthetic RNA molecules of various length and various GC contents which can be added to the RNA preparation and sequenced along with the mRNA. They can thus be used as controls to normalize the data. The control experiments that we analyze here all include ERCC. In Fig. S1, we plot the normalized variance estimator as a function of average expression for real and ERCC genes. We notice that the ERCC genes have an increased variance, and are sometimes expressed at a much higher level than regular genes. In order to avoid contaminating our results with this peculiar behavior of ERCC genes, we exclude them from our analysis.

2 Sampling noise introduces strong additional variability

In order to highlight how sampling noise introduces variability, we simulated expression values for 2361 cells using a distribution composed of two Gaussians in Seurat scale ($log(1 + 10^4\lambda_g)$). The first Gaussian had a mean of 0.75 and a scale of 0.25 and the second one had a mean of 2 and a scale of 0.2. We display on Fig. S2 this distribution as a blue line as well as the histogram of the simulated values in light blue. We observe that they are very close. We then simulate the sampling noise using this simulated expression and N_i values found in an existing scRNA-seq dataset (see Methods: Simulation of data). This simulation gives a number of count for this gene ($k_{i,g}$) for each cell. We then plot the histogram of the normalized expression ($log(1 + 10^4(k_{i,g}/N_{i,g})$) in dark red. We see the appearance of a proportion of cell with no detected expression as well as a general increased variability compared to the original distribution. To summarize this data, we also use a kernel density estimate in red (see Methods: Data representation for more details). This representation is used in Fig. 2, 3 and 7C, Fig. S3 and S7C. The Fig. S2 also shows that the overdispersion observed in the 'Density from data' curve compared to the 'Original distribution' is not due to oversmoothing of the kernel density estimate.

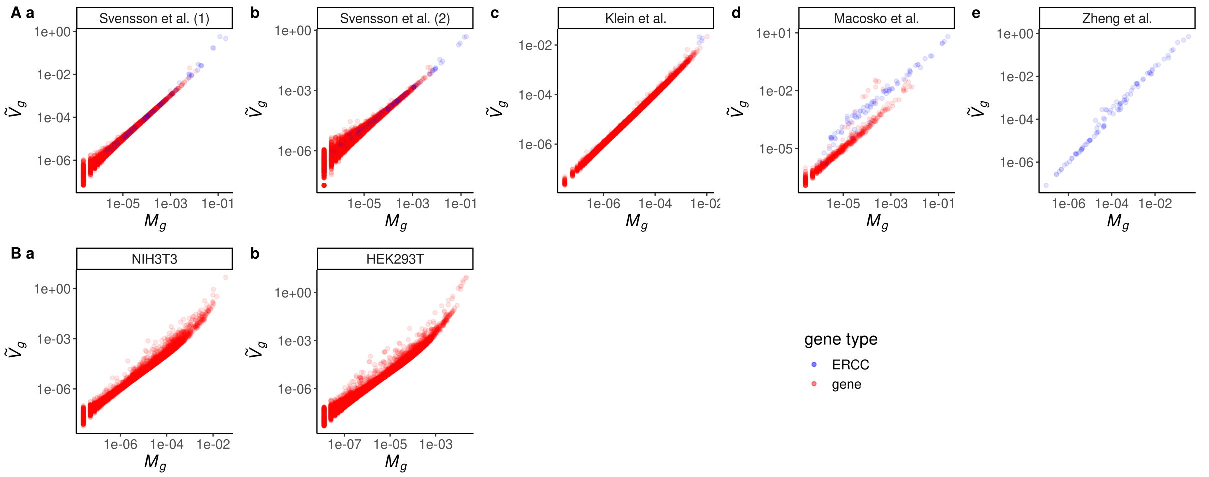

Fig. S1: ERCC exhibit an increased variance compare to mRNA coding for genes. In each data set, control data set (**A**) or real single-cell experiment (**B**), the normalized estimator of variance is plotted as a function of the estimated mean expression. Each dot is a gene. The dots are colored by gene type, spike-in ERCC (External RNA Control Consortium) are depicted in blue while mRNA are depicted in red.

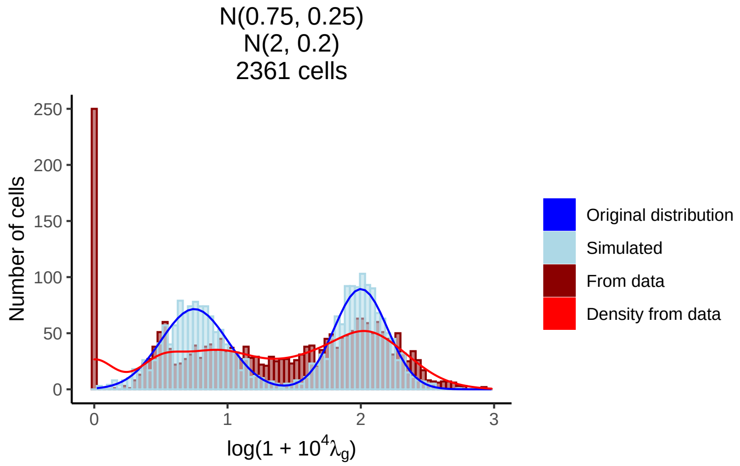

Fig. S2: Sampling noise introduces strong additional variability. In blue is displayed the distribution of the original data. In light blue is the histogram of the random expression values simulated from the distribution. In dark red is displayed the histogram of the simulated sampling (count for each cell, k_i, g) normalized by the number of total count (N_i). In red is displayed the density from data like in Fig. 2 and 3.

3 Comparison between baredSC and Sanity

As presented in the main text, baredSC and Sanity share common features but they highly differ on objectives and versatility. We report in Table S1 the differences between the two approaches making a distinction between baredSC used with a single Gaussian or with any number of Gaussians.

Table S1: Comparison between Sanity, baredSC using a single Gaussian (baredSC 1 gauss) and baredSC using any number of Gaussian (baredSC).

	Sanity	baredSC 1 gauss	baredSC
Objective	Denoise	Describe intrinsic variability	
Number of Gaussians in PDF	1		m
Flexibility on priors	No	Can be implemented	
Possible transformation of λ_g	log	log, $log(1 + N_t\lambda)$, others can be implemented.	
Computation time for a thousand cells per gene	seconds	minutes to hours	

We compared more precisely the results obtained by the three methods (Sanity, baredSC with a single Gaussian, baredSC with up to four Gaussians) on the five simulations used in Fig. 3. Indeed, using Sanity outputs we can represent the data in three different ways. First, Sanity assume that the distribution of expression can be described by its mean and variance. Among Sanity outputs, one can get the expected value of the mean and an estimation of its error as well as the expected value of the variance. Using these values, we can deduce the inferred PDF approximated by a single Gaussian. The main result of Sanity is the matrix of the log transcription quotients (LTQs) which are the expected value for each cell and the error on these values. Using baredSC, we can also evaluate these expected values and error (see Methods: Posterior per cell).

While the implementation and the priors differ between Sanity and baredSC with a single Gaussian, they give very similar results (Fig. S3). Interestingly, the second and third rows highlight how the representations using either the expected value for each cell or these expected values and their error bars affect the quality of the distribution when it is not a single Gaussian.

4 The number of samples required for convergence of MCMC increases with the number of Gaussians

For each seed used to run baredSC displayed in Fig. 4, the number of samples required to reach convergence is plotted in Fig. S4. We see that the model with a single Gaussian always reached convergence with 100'000 samples. Similarly, the model which corresponds to the number of Gaussians in the real distribution (dotted vertical line) nearly always converges with 100'000. On the contrary, the model with 3 or 4 Gaussians often require million(s) of samples to reach convergence. This is why the convergence using one to four Gaussians could not be reached within 30 hours for 625'000 and 3'125'000 cells in some of the generated distribution.

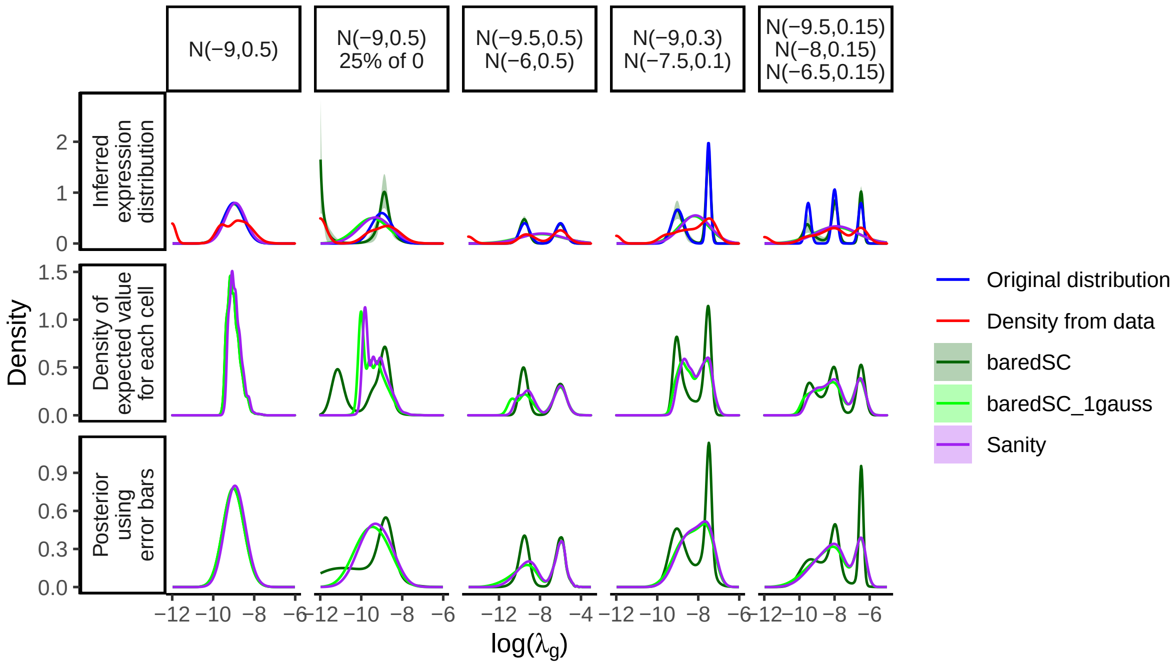

Fig. S3: Sanity and baredSC with a single Gaussian gives very similar results. Each column corresponds to a simulation. For each of them, the simulated distribution is plotted in blue in the first row (Inferred expression distribution) and its characteristics are written above (N for normal followed by mean and scale values in log as well as the proportion of cells with no expression). In the first row, the values obtained after Poisson simulation are summarized by the red curve (Density from data). The mean PDF obtained using baredSC with up to 4 Gaussians is depicted in dark green and the dark green area shows the quantiles at 16-84%. Similarly the results obtained using baredSC with a single Gaussian are plotted in light green. The results obtained using Sanity (using the estimation of the average and the variance of the distribution) are in purple. For the 3 models, the expected value was computed for each cell and the density of these values are plotted in the second row. Finally, the posterior using the expected value and the error for each cell is displayed on the last row.

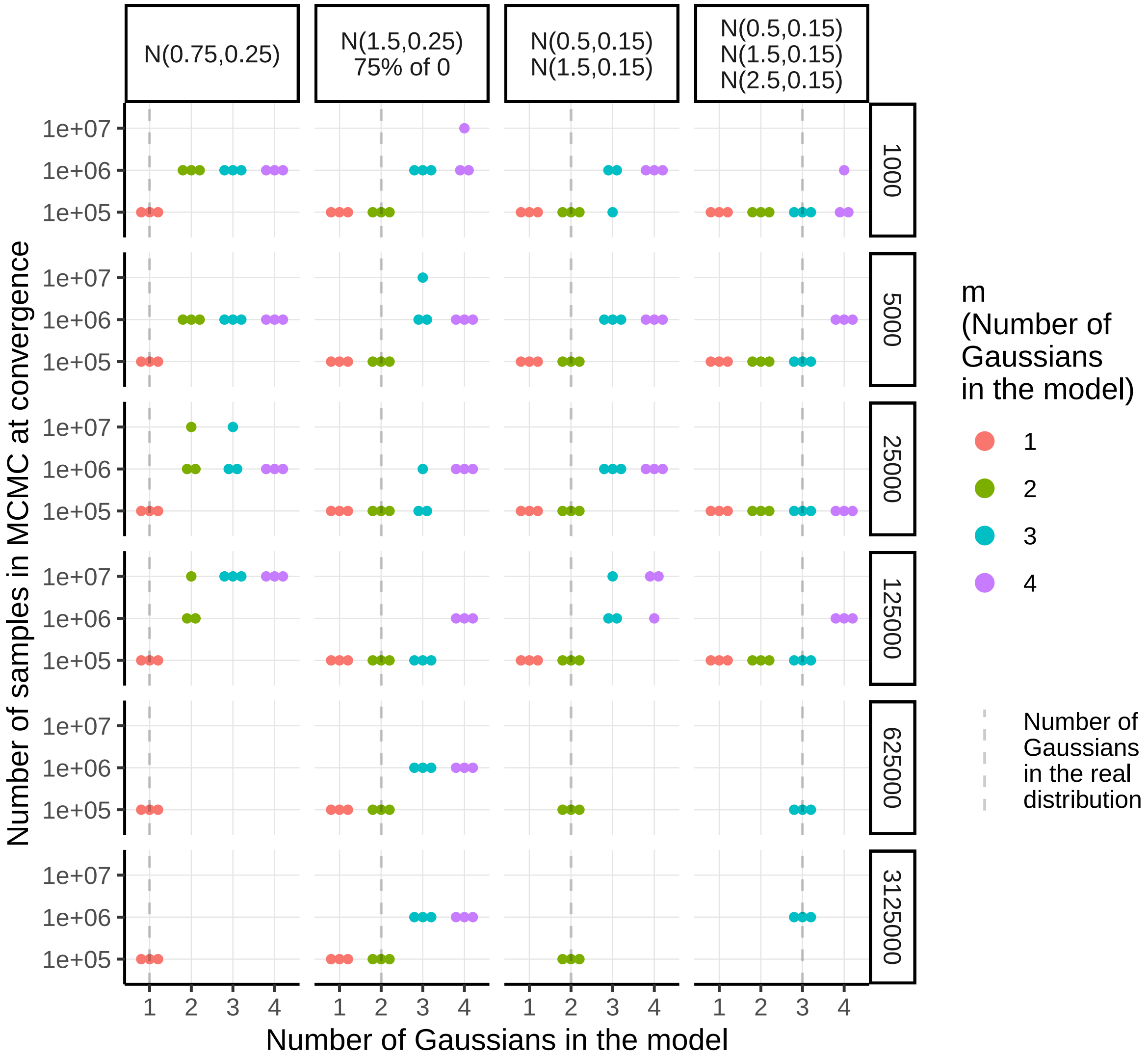

Fig. S4: Number of samples required for convergence of MCMC. Each column corresponds to an original distribution, each row corresponds to the number of cells simulated, each dot corresponds to a run of baredSC which converged before 30 hours. baredSC was run with 100'000 samples and the number of samples was multiplied by 10 until convergence is reached or job lasts longer than 30 hours. The vertical dotted line highlight the number of Gaussians expected for the original distribution.

5 baredSC in 2 dimensions performs well even with smaller number of cells

While in the main figure, all simulations were run for the whole set of cells (2361), we also run baredSC when only a smaller number of cells was provided (Fig. S5). With 1561 cells, the results are very close to the results obtained for the 2361 cells. The only exception is the case of each gene expressed in half independent (Fig. S5C) where it detects a significant but very modest correlation (0.09). For the smaller numbers of cells (300 and 500), it globally performs very well especially in simulations with multi-modal distributions. In Fig. S5A where the expression is very low, the algorithm fails to detect most of the (anti-)correlations but the average PDF is close to the simulated one. In this range of expression, a high number of cell is necessary to infer a good correlation of the distribution.

6 Comparison between baredSC and Sanity in two dimensions

Sanity evaluates the expected value of the log transcription quotients (LTQs) for each cell and each gene. Therefore, we can compare the results obtained by Sanity in 2 dimensions with baredSC results, especially for the evaluation of the correlation coefficient. We simulated values similarly to what has been done for Fig. 5 except that the normal distributions were simulated in the log scale (Fig. S6).

We can see that in the first two simulations where the original distribution is a single Gaussian with a weak (anti-)correlation, the correlation coefficient estimated by Sanity using the expected values (fourth row) is less than half the real correlation coefficient. This is in contrast with baredSC results (third row) which give better estimation. When using the posterior distribution from Sanity (last row), the (anti-)correlation is nearly undetected. In the third simulation where the original distribution is a single Gaussian without any correlation (independent), the posterior from Sanity gives very good results.

When two populations were simulated (last four simulations), we can distinguish the first two where the two Gaussians are well separated from the last two where they are very close. In the first case, Sanity outputs allow the identification of the two populations but the difference in the scales of the Gaussians cannot be distinguished. In these cases the correlation coefficients are reduced by 30% compared to the coefficients in the original distribution. In the last two cases, the Sanity results do not allow to separate the two populations and the correlation coefficients are about half the value in the original distribution.

In conclusion, Sanity can help to detect correlation or anti-correlation between genes but the correlation coefficient may be underestimated. In contrast, baredSC allows to retrieve very close confidence interval for the correlation coefficient and the inferred PDF is close to the original one.

7 Analysis of distribution of *Pitx1* expression in log axis

In baredSC, we implemented two scales. The scale used in the Seurat and Scanpy package which is $\log(1 + N_t X_{i,g})$ (with $N_t = 10^4$ in the Seurat package) and the regular log scale ($\log(X_{i,g})$). In all main figures of this article except Fig. 3, we used the Seurat scale. This choice is motivated by the decreased degeneracy of models in Seurat scale for cells with no count compared to the log scale. However, the log scale may be more convenient in some cases.

In particular, we use it in Fig. 3, Fig. S3, S6 and Fig. S7 to compare to other techniques which use the log scale. In the case of *Pitx1* expression, we have fluorescence levels from flow cytometry which are conventionally represented in log scale (Fig. S7A). We use baredSC to retrieve the PDF in log scale

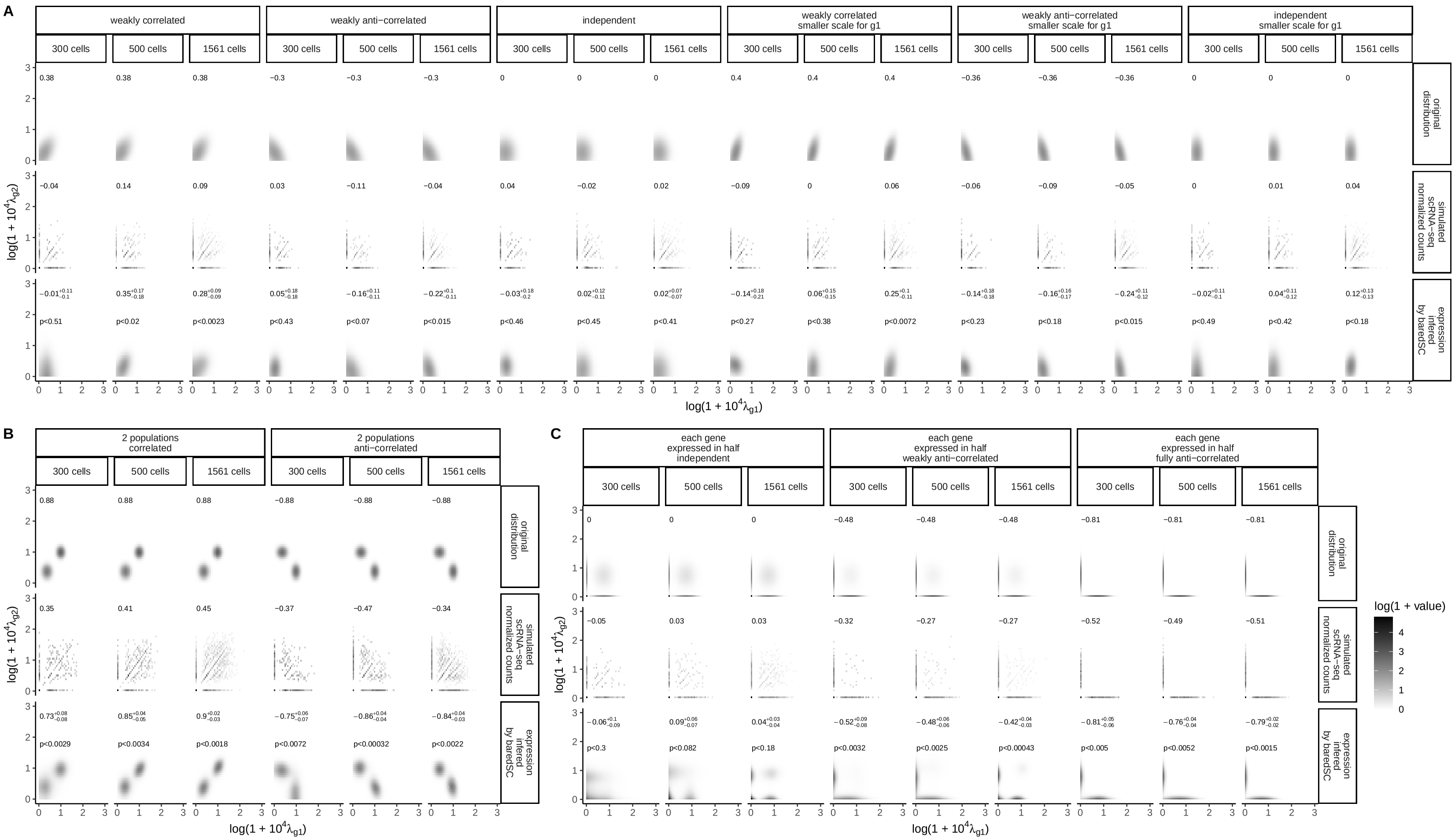

Fig. S5: baredSC in 2D with smaller number of cells. This figure is identical to Fig. 5 except that the result for each group of cells is plotted.

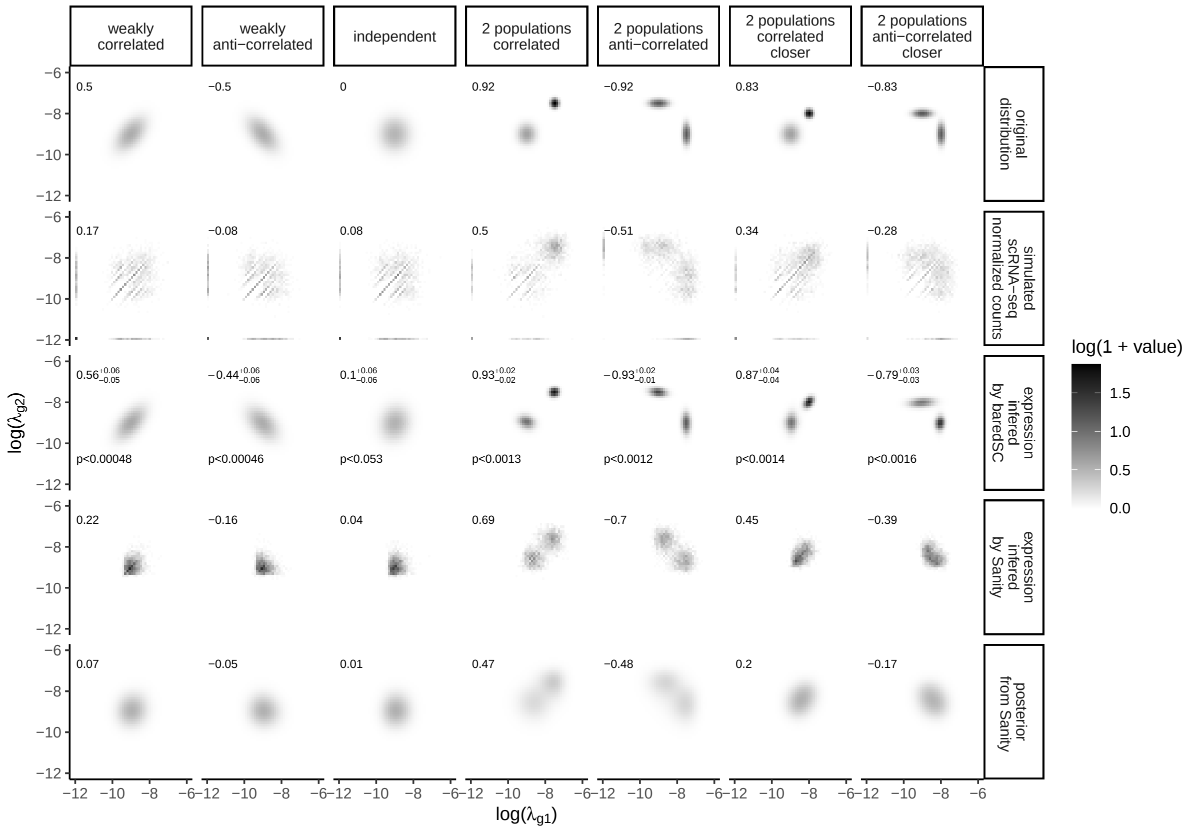

Fig. S6: baredSC better estimates correlation coefficients compared to Sanity. In each simulation, the PDF of the original distribution is plotted in the top row. The values obtained after Poisson simulation are plotted in the second row. The mean PDF obtained by baredSC is plotted in the third row. The Sanity values are plotted in the fourth row. On the last row is plotted the PDF obtained using the posterior distribution from each cell using Sanity values and error bars. In the top left corner is written the Pearson's correlation coefficient and for baredSC its confidence interval. For baredSC, in the bottom left corner is written the one-sided p-value.

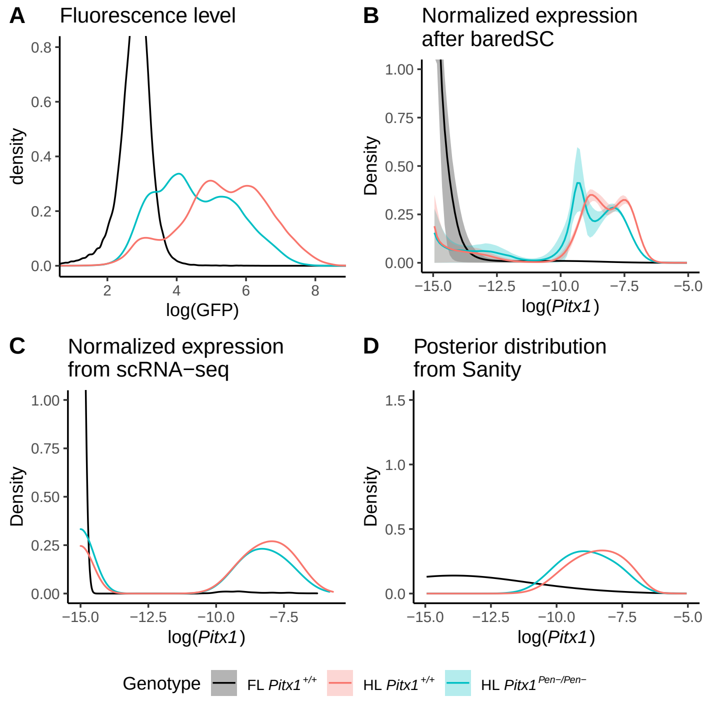

Fig. S7: baredSC retrieves the trimodal distribution of *Pitx1*. **A**: Distribution of fluorescence intensity obtained by flow cytometry in regular log scale. **B**: Distribution of normalized expression from scRNA-seq as provided by baredSC when run in log scale. **C**: Distribution of normalized expression from scRNA-seq with a regular log scale. **D**: Posterior distribution obtained with Sanity.

(Fig. S7B). For FL *Pitx1*$^{+/+}$ (in black), where *Pitx1* is not expressed, we observe that the distribution is concentrated on the low values. However, we observe a larger confidence interval compared to the distribution obtained in the Seurat scale (Fig. 5D). This is due to the degeneracy of the model at low value of expression: having no count is similarly probable for expression values of -13 in log scale (2e-6 which is much smaller than $1/N_i$) or below. Thus, one should consider the low expression range as cells that do not express *Pitx1*. As explained in the article, in this range of expression, it is difficult to compare both techniques as the flow cytometry has a high level of background. At higher level of expression (right part), the PDF of HL *Pitx1*$^{\textit{Pen-/Pen-}}$ and HL *Pitx1*$^{+/+}$ have a highly similar shape between the flow cytometry (Fig. S7A) and the baredSC results (Fig. S7B). These results confirm what we observed in Fig. 5 that the fluorescence level is highly correlated to the level of expression of *Pitx1*. We then compared these results to the density plot of normalized expression (Fig. S7C). For this representation of the normalized counts, the expression of cells with no expression was artificially put to the minimal value. We see that the PDF of cells where *Pitx1* is detected is close to a Gaussian for both HL samples. We also run Sanity to visualize the posterior distribution given by this algorithm (Fig. S7D). We see that the tissue which does not express *Pitx1* (FL *Pitx1*$^{+/+}$ in black) is described as a large Gaussian, suggesting that a fraction of cells expresses *Pitx1* at low level. The PDF of samples which express *Pitx1* (HL in blue and red) is unimodal. This show that the posterior distribution from Sanity is not able to detect the trimodal expression of *Pitx1* in HL samples.
